# Inhibition of Inflammatory Changes in Human Myometrial Cells by Cell Penetrating Peptide and Small Molecule Inhibitors of NFκB

**DOI:** 10.3389/fimmu.2018.02966

**Published:** 2018-12-20

**Authors:** Leo R. I. Gurney, Julie Taggart, Wing-Chiu Tong, Arwyn T. Jones, Stephen C. Robson, Michael J. Taggart

**Affiliations:** ^1^Institute of Genetic Medicine, Newcastle University, Newcastle upon Tyne, United Kingdom; ^2^School of Pharmacy and Pharmaceutical Sciences, Cardiff University, Cardiff, United Kingdom; ^3^Institute of Cellular Medicine, Newcastle University, Newcastle upon Tyne, United Kingdom

**Keywords:** preterm birth, nuclear factor kappa B, cell penetrating peptide, tocolytic, myometrial cell

## Abstract

Complications arising from Preterm Birth are the leading causes of neonatal death globally. Current therapeutic strategies to prevent Preterm Birth are yet to demonstrate success in terms of reducing this neonatal disease burden. Upregulation of intracellular inflammatory pathways in uterine cells, including those involving nuclear factor kappa-B (NFκB), have been causally linked to both human term and preterm labor, but the barrier presented by the cell membrane presents an obstacle to interventions aimed at dampening these inflammatory responses. Cell penetrating peptides (CPPs) are novel vectors that can traverse cell membranes without the need for recognition by cell surface receptors and offer the ability to deliver therapeutic cargo internal to cell membranes. Using a human uterine cell culture inflammatory model, this study aimed to test the effectiveness of CPP-cargo delivery to inhibit inflammatory responses, comparing this effect with a small molecule inhibitor (Sc514) that has a similar intracellular target of action within the NFκB pathway (the IKK complex). The CPP Penetratin, conjugated to rhodamine, was able to enter uterine cells within a 60 min timeframe as assessed by live confocal microscopy, this phenomena was not observed with the use of a rhodamine-conjugated inert control peptide (GC(GS)_4_). Penetratin CPP conjugated to an IKK-inhibitory peptide (Pen-NBD) demonstrated ability to inhibit both the IL1β-induced expression of the inflammatory protein COX2 and dampen the expression of a bespoke array of inflammatory genes. Truncation of the CPP vector rendered the CPP-cargo conjugate much less effective, demonstrating the importance of careful vector selection. The small molecule inhibitor Sc514 also demonstrated ability to inhibit COX2 protein responses and a broad down-regulatory effect on uterine cell inflammatory gene expression. These results support the further exploration of either CPP-based or small molecular treatment strategies to dampen gestational cell inflammatory responses in the context of preterm birth. The work underlines both the importance of careful selection of CPP vector-cargo combinations and basic testing over a broad time and concentration range to ensure effective responses. Further work should demonstrate the effectiveness of CPP-linked cargos to dampen alternative pathways of inflammation linked to Preterm Birth such as MAP Kinase or AP1.

## Introduction

Preterm birth, or birth before 37 weeks completed gestation as defined by the World Health Organization, is the main cause of neonatal death in developed countries and presents an enormous global problem (1). Being born too soon can confer significant clinical deficits throughout life, leading to neuro-developmental disorders such as cerebral palsy, learning impairment and visual disorders; with such problems being more likely to occur with greater frequency and severity at earlier gestations of birth (2).

Preterm birth is a syndrome best understood as the final endpoint of several possible pathophysiological events. It can be initiated by an array of processes including: uterine over-distension, utero-placental hemorrhage or ischaemia, maternal stress, cervical insufficiency, and inflammation with or without clinically apparent infection (3). Parallel to this, there is evidence to suggest that the processes that drive spontaneous human labor, whether at term or preterm, have an inflammatory basis. Macrophage and neutrophil numbers in human fetal membranes, myometrium and cervix are increased in tissue derived from laboring patients (4, 5) and such invasion leads to increases in local cytokine and chemokine production including increases in IL1β, IL8, and TNFα (6). These changes may precipitate, or contribute to, a sequence of uterine activation processes leading to labor (7). Furthermore, invasion of the decidua by these cell types occurs in animal models of preterm birth and precedes the onset of labor (8); although whether this invasion is a cause or consequence of labor remains to be fully defined (9). The inflammatory milieu provoked within the uterine environment has been reported to upregulate varied pro-inflammatory pathways in uterine and placental cells including signaling pathways involving p38 MAP kinase (p38 MAPK) and Activator Protein 1 (AP1) (10, 11). Most importantly, upregulation of intracellular pathways involving the transcription factor nuclear factor κB (NFκB) are suggested to play a central role in the sequelae of inflammation-associated preterm birth (12).

NFκB proteins are a family of five structurally related transcription factors named p65 (Rel A), RelB, c-Rel, p50 (NFκB-1), and p52 (NFκB-2) present in nearly all mammalian cells that play a ubiquitous role in inflammatory and infectious responses (13). All NFκB proteins exist in the form of heterogeneous dimers, the most commonly described of which is p65/p50 which is known to activate the expression of pro-inflammatory genes within the uterus (14). Within the canonical NFκB pathway interaction between cell surface receptors and lipopolysaccharide (LPS), or pro-inflammatory cytokines such as IL-1β, TNFα, or IL6 activates the IkB kinase complex (IKK complex) (13). This kinase complex consists of two catalytic subunits (IKKα & IKKβ) each containing a six amino acid segment LDWSWL, known as the Nemo Binding Domain (NBD), which forms the basis of their binding site with the regulatory NFκB essential modulator (NEMO) scaffold protein. Activation of IKK leads to the targeted phosphorylation of IkBα allowing for the ubiquitination and subsequent degradation of this anchoring protein by the proteasome. This step allows it to translocate to the nucleus of the cell, a process enhanced by the IKK-dependent phosphorylation of p65. Once in the cell nucleus, NFκB binds to target gene promoters regulating the expression of inflammatory genes including those that transcribe IL1β, TNFα, IL6, IL8, CXCL2, MMP 9, and COX2 (15). Due to the presence of κB binding sites on the IκBα gene promoter, activation of NFκB leads to the rapid resynthesis of IκBα which dissociates NFκB from DNA complexes and is shuttled out of the nucleus in an inactive complex (16).

Amongst the evidence supporting the role of NFκB activation in labor are observations from *in vitro* studies on human myometriaI cells: activation of NFκB has been shown to promote the expression of the inducible prostaglandin synthase enzyme cyclooxygenase 2 (COX2) leading to subsequent increases in prostaglandin production in these cells (17). Prostaglandins E2 and F2α promote uterine contractions and their increased production within reproductive tissues is associated with the onset of human labor (18), thus increases in COX2 expression are thought to correspond to both inflammatory and contractile responses in the myometrium during human labor.

Myometrial cell NFκB activation also promotes the increased production of pro-inflammatory cytokines including IL-6 and IL-8 (19), matrix metalloproteinases (20), and up-regulates the expression of mRNA encoding genes associated with labor including the oxytocin receptor and gap junction proteins (21, 22).

Agents aimed at the acute prevention of preterm birth are a class of drugs referred to as tocolytics. Despite their use in more than 3000 clinical trials over 60 years, tocolytic agents have yet to demonstrate significant improvements in neonatal outcome and their use is frequently associated with an unacceptably high frequency of unwanted sequelae (23). This leaves an urgent need for the exploration of new therapeutic strategies aimed at targeting the molecular pathways whose upregulation is linked to preterm birth.

Peptides targeting protein-protein interactions that regulate cellular processes are gaining increasing traction as therapeutic entities that target a number of diseases. As biological molecules they offer very high selectivity and specificity and are relatively cheap to manufacture (24) A major barrier to the development of new peptides as pharmaceuticals is presented by the cell membrane: the lipid bilayer can prevent the passage of therapeutics from extracellular space to intracellular targets that often lie within the cytosol of a cell. To overcome this obstacle requires a vector system that can deliver cargo to the cell cytosol either directly through the plasma membrane or through utilizing endocytosis as a portal to cytoplasm before mediating endocytic escape processes to reach the cytosol (25).

Cell Penetrating Peptides (CPPs) offer an attractive solution to this drug delivery puzzle: they are characterized as short peptides, usually < 30 amino acids length, that have the ability to cross cell membranes without the need for recognition by cell surface receptors (26). CPPs have been shown to deliver cargo efficiently at low doses to a diverse range of cell types and a number of studies in varied clinical fields have confirmed the potential of CPP-cargo conjugates as therapeutic agents (27). This has led to several CPP-based therapies being tested in phase 3 clinical trials for a diverse range of inflammatory conditions (28). Despite this, the effectiveness of CPP-linked therapy in gestational cells has yet to be examined in detail.

Amongst the broad array of cargoes that can be conjugated to CPPs and delivered intracellularly are molecules with the capability to block NFκB-dependent signaling (15). The best described CPP-cargo conjugate with NFκB inhibitory ability is the Nemo Binding Domain (NBD) peptide: an 11 amino acid residue peptide which was designed to span the NBD and therefore disrupt the interaction between the three IKK subunits within the NFκB canonical pathway, with the effect of inhibiting the inflammatory ligand-dependant activation of NFκB (29). The NBD peptide, conjugated to varied CPP vectors, has been shown to down-regulate elicited NFκB responses and thus improve physiological endpoints of inflammation both *in vitro* and in animal models of diseases as varied as Muscular Dystrophy and Parkinson's disease (30–32).

Small molecule inhibitors targeting several levels of the NFκB pathway have been reported including those blocking the phosphorylation of IkBα via IKK complex inhibition or preventing proteasome degradation of this molecule (33). Inhibitors have also been demonstrated that prevent NFκB localization to the nucleus or block post translational modifications of p65. The advantages of using small molecule drug inhibition include low immunogenicity and the possibility of attaining high oral bioavailability with the aim of inhibiting specific signaling pathways. However, high concentrations may be required to achieve therapeutic benefit; they may be poor at crossing biological barriers, and are often substrates for swift removal via drug efflux proteins (34).

The non-peptidic inhibitor Sc514 represents an established small molecule compound targeting NFκB mediated effects. It exerts action via inhibition of the IKKβ portion of the IKK complex and therefore putatively offers discrete inhibition of the NFκB pathway via a mechanism similar to the NBD peptide (35). It has demonstrated ability to significantly reduce LPS-stimulated TNFα secretion in placentally-derived human primary cells (36), however it has yet to be broadly tested in a uterine cell setting. Thus, the use of this drug allows examination of the efficacy of a small molecule approach to inhibit NFκB-related inflammatory responses in myometrial cells, as well as offering a comparison with the CPP-cargo inhibitory approach presented by the NBD peptide.

With the intention of broadening the scope of anti-inflammatory candidate agents available to be tested for their potential as treatments for preterm birth, the aims of this study were to: (i) first define the ability of CPPs to enter human myometrial cells and deliver the NBD peptide and (ii) subsequent to this, examine the effectiveness of CPP-NBD peptide conjugates in dampening NFκB related signaling compared to Sc514 in human myometrial cells.

## Materials and Methods

### Subjects & Samples

Ethical approval was obtained from Newcastle and North Tyneside Research Ethics Committee (10/H0906/71) to perform research on samples collected as part of the Newcastle Utero-placental Tissue Bank. Human myometrial tissue was obtained from patients following written informed consent from non-laboring women with uncomplicated, singleton pregnancies undergoing elective Cesarean section at term (≥37 weeks gestation). Myometrial muscle strip biopsies approximately 1 × 1 cm in size were taken from the upper portion of the lower segment uterine incision and placed in tissue collection buffer.

Biopsies were excluded from women with underlying medical or obstetric disease, women on any current medication, those with a body mass index outside range 20–35 kg/m^2^, or who gave birth to a baby with weight below the 10th percentile or above the 90th percentile. Separate myometrial biopsies from 46 patients were included in the study.

### Preparation of Myometrial Cells

Human myometrial biopsies were micro-dissected under a light microscope to isolate myometrial tissue from any remaining decidua. In a microbiological safety cabinet, tissue was cut into small fragments before adding 10 mls warmed hanks balanced salt solution containing 10 mg each of collagenase 1A and XI (Sigma Aldrich, C7657, C9891) plus 20 mg bovine serum albumin (Sigma Aldrich, A6003). This tissue digestion mix was placed in an orbital shaker at 110 rpm for approximately 40 min at 37°C. The sample was then triturated, filtered through a 70μm cell strainer into 10ml warmed media and centrifuged at 1,000 rpm (89 × g) for 5 min. The supernatant was discarded and cell pellet re-suspended in GlutaMAX cell culture media (Life Technologies 61965) containing 10% FCS and Penicillin/Streptomycin until 80–90% confluent (37).

### Cell Penetrating Peptides

CPP's were custom synthesized commercially and purchased from either EZ Biolabs (USA) or Abingdon Health Laboratory (UK) services. Rhodamine fluorophore cargo (excitation 561 nm, emission 617 nm) was labeled at the N-terminal end of either Penetratin or Pen(43-56) CPP. NBD or inactive NBD mutant cargo was conjugated at the C-terminal end of either Penetratin or Pen(43-56) CPP. Aminohexanoic acid (Aca) was used as a linker between CPP and cargo. The amino acid structures of the peptides used in experimentation are detailed in Table 1.

**Table 1:** The nomenclature and amino acid structure of peptides used for experimentation.

**Peptide name**	**Structure**
Rhodamine labeled Penetratin	Rhodamine–RQIKIWFQNRRMKWKK
Rhodamine labeled Pen-NBD	Rhodamine-RQIKIWFQNRRMKWKKTALDWSWLQTE
Rhodamine labeled Pen(43-56) – NBD	Rhodamine-RQIKIWFQNRRMKW-Aca- TALDWSWLQTE
Rhodamine labeled GS_4_(GC)	Rhodamine - GSGSGSGSGC
Pen-NBD	RQIKIWFQNRRMKWKKTALDWSWLQTE-NH2
Pen-NBD Mutant	RQIKIWFQNRRMKWKKTALDASALQTE-NH2
NBD	TALDWSWLQTE
Pen (43-56)-NBD	Ac-RQIKIWFQNRRMKW-Aca-TALDWSWLQTE-NH2
Pen (43-56)-NBD Mutant	Ac-RQIKIWFQNRRMKW-Aca-TALDASALOTE-NH2

*Rhodamine fluorophore cargo (excitation 561 nm, emission 617 nm) is labeled at the N-terminal end of Penetratin or Pen(43-56) CPP. NBD or inactive NBD mutant cargo is conjugated at the C-terminal end. Acyl (Ac), Aminohexanoic acid (Aca), Amide (NH2)*.

To test specificity of COX2 responses to the NBD peptide, the effect of a Penetratin-conjugated mutant version of the NBD peptide: Pen NBD (Mut), was examined. This peptide contains the substitution of two tryptophan (W) amino acid residues to alanine (A) thus rendering it unable to span the NBD and interfere with IKK related signaling toward NFκB (29).

To further investigate the importance of CPP vector structure for the delivery of biologically active cargo a truncated form of Penetratin CPP was conjugated to the NBD peptide: Pen(43-56)-NBD (38).

### Live Cell Confocal Microscopy

Primary human myometrial cells at passage P ≤ 4 were transferred into 4 or 8-well microscope Ibidi μ-Slides (Thistle Scientific, IB-80441, IB-80821) and grown up to 80–90% confluency. Cells were washed in phosphate buffered saline and changed to serum-reduced media (0.1%FCS) for 18–24 h (39). Cells were then loaded with 1 μM Hoechst nuclear dye (Life Technologies, 33342) for 1 h and washed with PBS before transfer to the confocal microscope where they were maintained within a temperature-controlled chamber maintained at 37°C, 5%CO_2_ on the stage of the inverted microscope.

Initial images were collected to assess baseline cellular auto-fluorescence prior to the addition of varying concentrations of fluorophore-CPP/fluorophore- CPP-Cargo/fluorophore-control peptide combinations (see Table 1) that were ready made up in serum-reduced cell media. Further images were captured 60 min following application. During imaging cells were placed in phenol red free Dulbecco modified eagle medium (Life Technologies, 21063) to reduce background glare.

To make a thorough assessment of fluorescent uptake throughout whole cells, all images were captured as a series of slices in the Z plane (range 3–10 slices per area selected, each slice 0.54 μm apart) with the 40x objective using live cell spinning disk confocal microscopy (Andor Revolution XD coupled to an Olympus IX-81 inverted microscope; Andor Belfast UK). All images were digitally recorded with IQ2 software (Andor, Belfast, UK).

### Cell Stimulation Experiments for Subsequent Protein or mRNA Extraction

Primary human myometrial cells at passage ≤ P4 were split equally between 6 or 12 well-plates and grown to 80–90% confluency. At this point media was changed to serum-reduced (0.1% FCS) media for 18 to 24 h before discarding from each well. Five hundred microliters of fresh media (0.1% FCS) was produced containing: Pen-NBD, Pen(43-56)-NBD, Sc514 (Tocris bioscience, 3318); or controls (equivalent volume DMSO, Pen-NBD Mutant, NBD alone) at indicated concentrations. The inhibitor/control media was added to wells as a pre-incubation step. After 1 h, 10 ng/ml IL1β (Peprotech, 200-01B), or equivalent volume DMSO vehicle, was added to each well.

For western blotting experiments cells were washed with PBS before being lysed using sucrose cell lysis buffer (62.5 mM Tris-HCl pH6.8, 2% SDS, 10% saccharose) containing 20 μl/ml protease inhibitor (Sigma Aldrich, P1860) and 5 μl/ml phosphatase inhibitor (Sigma Aldrich P2745) at times of 0/15/60/120/240 minute from cytokine stimulation.

For RNA array experiments, 4 h following cytokine stimulation cells were washed with PBS before RNA was extracted using the RNeasy Mini Kit (Qiagen, 74101) according to the manufacturers' protocol.

### Western Immunoblotting

Cell lysate samples were sonicated and underwent Lowry assay to determine overall protein concentration before dilution in an equal volume of 2x Laemmli buffer (Tris pH6.8 250 nM; SDS 4% w/v; Glycerol 10% v/v; β-mercaptoethanol; bromophenol blue). Ten micrograms of sample protein per lane was loaded onto a 1.5 mm 10% sodium dodecyl sulfate poly-acrylamide gel (SDS-PAGE) before undergoing separation by electrophoresis, gels then underwent electrophoretic transfer to a methanol-activated polyvinylidine difluoride (PVDF) membrane.

Following a blocking step of washing membranes in 5% non-fat dry milk in Tris-buffered saline with 0.1% Tween-20 (TBS-T) for 1 h at room temperature, membranes were incubated overnight at 4°C with primary anti-COX-2 antibody 1:500 (Cayman laboratories, CAY160112), anti-IκB-α antibody 1:500 (Santa Cruz, C21 Sc371) anti-phospho-P65 antibody 1:2000 (Cell Signaling, 3013), or anti-P65 antibody 1:15,000 (Santa Cruz, Sc372) in 1% non-fat dry milk with TBS-T. After three washing steps with TBS-T, secondary antibody incubation occurred for 1 h at room temperature. Polyclonal horseradish peroxidise conjugated goat anti-mouse immunoglobulin 1:3000 (DAKO, P0447) in 1% non-fat dry milk was used in conjunction with COX2 antibody and a polyclonal horseradish peroxidise conjugated goat anti-rabbit immunoglobulin 1:5000 in 1% non-fat dry milk (DAKO, P0448) was used for IκB-α, phospho-P65 and P65 antibody.

After further TBS-T wash, enhanced chemiluminescent (ECL) reagent (Fisher Scientific, 12316992) was applied to the PVDF membranes for 5 min. Membranes were dried, placed in development cassettes and developed manually onto photographic film in a dark room. Equal loading of proteins was assessed by staining of the PVDF membrane with Napthol Blue Black Reagent (0.1% napthol blue black, 10% methanol, 2% acetic acid) to detect actin protein (43kDa). Developed films were densitometrically scanned using UMAX PowerLook III (UMAX) and quantification performed with Bio Image Intelligent Quantifier 2 software (Bioimage systems).

### Measurement of mRNA Expression

Following extraction of RNA (as per above), gene expression was assessed via the measurement of mRNA transcripts across a panel of selected genes using RT qPCR arrays (Qiagen, Custom RT2 profiler PCR arrays, 330171). All work was carried out in a ribonuclease free environment with ribonuclease free equipment. Measurement of the RNA content in each sample was undertaken using a nanodrop spectrophotometer (ND-1000, Labtech), with suitable RNA purity considered to be a 260/280 ratio of >2.1. 0.5 μg of total RNA from each sample was added to the genomic DNA elimination mix from RT2 first strand kit (Qiagen, 330404) reverse transcription was then carried out according the manufacturers' instructions.

cDNA as synthesized from 0.5 μg of total RNA from each original sample was added to the SYBR Green Master Mix (Qiagen, 330171). Twenty-five microliters of this final mix was then added to each well of a 96 well-array plate, with each well-containing a different primer for a gene of interest or control. The plate was sealed with optical film, centrifuged at 1,000 rpm (89 × g) for 1 min before inserting the plate into the PCR cycler (Step One Plus, Applied Biosciences) according to a protocol of enzyme activation hot start at 95°C for 10 min, followed by 40 cycles of denaturing at 95°C for 15 s and extension at 60°C for 60 s.

Fluorogenic data was collected via the FAM channel and the cycle threshold (Ct) values were calculated by applying a threshold limit that represented the exponential phase of amplification. To ensure comparability of gene expression between different array plates the same threshold limit was applied to all experiments (Ct 0.116). This was calculated as a mean threshold value from the first 6 arrays and corresponded to the exponential phase of the amplification curve for all subsequent experiments.

These array plates were created bespoke for the authors by Qiagen and the genes selected for examination are demonstrated in Table 2. The approach taken to gene selection is described within the results section below.

**Table 2:** Genes used in RT2 profiler PCR array plates.

**Gene grouping**	**Gene name**
Labor associated genes	OXTR/MMP 9/ MMP19/ TIMP1/ GJA1/GJB2
Inflammatory Genes	IL1A/IL1B/TNFA/IL6/IL8/ICAM1/ SOCS3 /IL1R1/IL1R2/IL4R/CXCL2/ CXCL1/CXCL6/CCL2
NFκB pathway	NFKB1/NFKB2/ RELA/NFKBIA/NFKBIZ
Prostaglandin production	PTGER3/PLA2G2A/ PTGES/PTGS2
G protein receptors	GPR37/GPR34/RGS 10
Novel genes	S100A9/ S100A8/STAT1/ FOXO1/ ZEB1/ LILRA5/ SPINK5/FOSB/JUN/TRIB1
Housekeeping genes	GAPDH/ACTB/B2M

Analysis of mean Ct values and quality control were evaluated by exporting mean Ct values for each gene on the RT2 array plate to the following data analysis website supported by Qiagen: https://dataanalysis.sabiosciences.com/pcr/arrayanalysis.php.

Gene expression was normalized to three reference housekeeping genes β2-microglobulin (B2M), GAPDH and ACTB, and results calculated using the ΔΔC_T_ method.

For all qPCR gene array experiments raw Ct values are included in the Supplemental Data 1.

### Statistical Analysis

Prism 7.0 (GraphPad) software was used to perform statistical analysis. For western blotting data statistical analysis was performed on raw optical densitometry values. One or two-way (time/dose) ANOVA with *post-hoc* corrections were performed to compare differences between groups, as indicated in figure legends. For initial mRNA expression experiments comparing untreated and IL1β treated samples (Figure 8) *P*-values were calculated based on a Student's *t*-test of the replicate 2^∧^ΔΔCt values of each gene. For subsequent mRNA experiments comparing inhibitor groups (Table 3 and Supplemental Figure 3) one-way ANOVA with Bonferroni *post-hoc* corrections were performed to compare differences between groups. Statistical significance was assumed at *P* < 0.05.

**Table 3:** Effect of CPP-based or small molecule inhibitors on IL1β induced gene expression changes in myometrial cells.

**Gene group**	**Gene name**	**Inhibitor**		
		**Pen-NBD**	**Pen(43-56)-NBD**	**Sc514**
Labor associated genes	OXTR	↓ (*p* = 0.01)	–	–
	MMP9	–	–	↓ (*p* = 0.01)
Inflammatory cytokines	IL1A	↓(*p* ≤ 0.0001)	↓(*p* = 0.003)	↓(*p* = 0.001)
	IL1B	↓(*p* ≤ 0.0001)	↓(*p* = 0.034)	↓(*p* = 0.004)
	TNF	–	–	↓(*p* = 0.0004)
	IL16	↓(*p* = 0.016)	–	↓(*p* = 0.0004)
	IL18	↓(*p* = 0.023)	–	↓(*p* = 0.0007)
	ICAM1	–	–	↓(*p* = 0.04)
Inflammatory chemokines	CXCL2	–	–	↓ (*p* = 0.0013)
	CXCL6	↓(*p* ≤ 0.0001)	↓(*p* ≤ 0.0001)	-
	CCL2	↓(*p* ≤ 0.0001)	↓(*p* = 0.0003)	↓(*p* = 0.01)
NFκB family genes	NFKB1	↓(*p* = 0.0039)	–	↓(*p* = 0.001)
	NFKB2	↓(*p* = 0.01)	–	↓(*p* = 0.045)
	RELA	↓(*p* = 0.0049)	–	-
	NFKBIZ	–	–	↓ (*p* = 0.008)
Prostaglandin pathway genes	PTGES	↓(*p* ≤ 0.0001)	↓(*p* = 0.003)	–
	COX2	↓ (*p* = 0.0007)	–	↓(*p* = 0.01)

*Taken from the 42 gene array, the table demonstrates only the genes with significantly altered expression patterns between control (IL1β alone) or treatment (IL1β plus either 100 μM Pen-NBD/100 μM Pen(43-56)-NBD/50 μM Sc514) groups. ↓ indicates significantly altered gene expression in presence of inhibitor compared with IL1β alone. – indicates no significant change between groups. Scatter graphs demonstrating changes for each gene displayed can be found in Supplementary Figure 3*.

For all experimental conditions: each n represents a biological replicate i.e., each n is a separate experiment using cell cultures grown from myometrial biopsy of a new patient.

## Results

### Penetratin CPP Effectively Delivers Fluorescent Cargo to Human Myometrial Cells

Using the well-characterized Penetratin CPP, we initially investigated whether human myometrial cells were accessible to the CPP-mediated delivery of rhodamine fluorophore.

**Figure 1:**
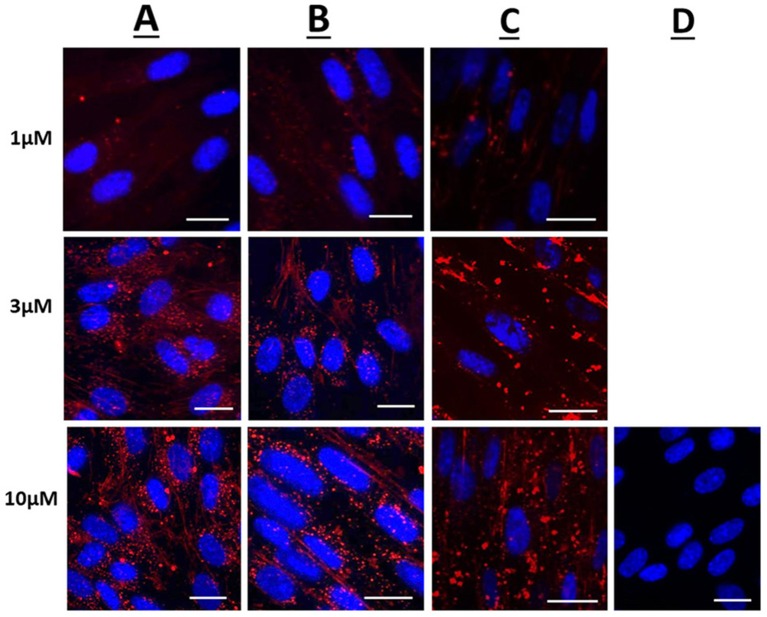
Confocal microscopy images of live human uterine smooth muscle cells following application of rhodamine-conjugated CPPs or CPP-cargo combinations. Images captured 60 min following addition of either 1, 3, or 10 μM concentrations of **(A)** Rhodamine—Penetratin, **(B)** Rhodamine—Pen-NBD, or **(C)** Rhodamine—Pen (43-56)-NBD. **(D)** Displays cells imaged 60 min following addition of 10 μM control peptide Rhodamine - GS4 (GC). Scale bars 20 μm. Cells are maintained in serum deprived media within a temperature-controlled chamber (37°C, 5%CO2). To demonstrate intra-cellularity of uptake, images are taken from the center of a Z-stack of 3–10 slices (0.54 μm apart) intended to capture the full depth of the cell. Images are representative of 3 independent experiments.

Figure 1 displays representative confocal microscope images of live uterine smooth muscle cells 1 h after application of rhodamine fluorescent cargo conjugated to either Penetratin (CPP), Pen-NBD, or to Pen(43-56)-NBD. The delivery of fluorescent cargo was assessed by comparison to that of a rhodamine-conjugated control peptide purported to not have cell penetrating ability [GS_4_ (GC)] (40) which can be viewed in Figure 1D.

At 1, 3, and 10 μM the rhodamine fluorophore was successfully delivered internal to the cells within 60 min of application, with an increase in intensity of uptake with increasing concentration (Figures 1A–C). By comparison the inert peptide GS_4_ (GC) at 10 μM did not appreciably deliver the rhodamine fluorophore after 60 min (Figure 1D). This indicates the ability of CPPs to deliver cargo to human myometrial cells.

Following 60 min of cellular application, the CPP-fluorophore conjugates (Figure 1A) and also the CPP-cargo-fluorophore conjugates (Figures 1B,C) largely demonstrated a punctate pattern of labeling indicative of endosomic labeling. This is best demonstrated in the image of Rhodamine-Pen-NBD at 10μM (Row B, third picture from left). It can be noted that this punctate labeling forms larger accumulations with the use of Pen(43-56)-NBD (Figure 1C), this may be attributed to alteration of the CPP-cargo amino acid structure, a phenomenon referred to in the discussion below.

### The CPP-Cargo Conjugate Pen-NBD Inhibits Cytokine-Activated COX2 Expression

**Figure 2:**
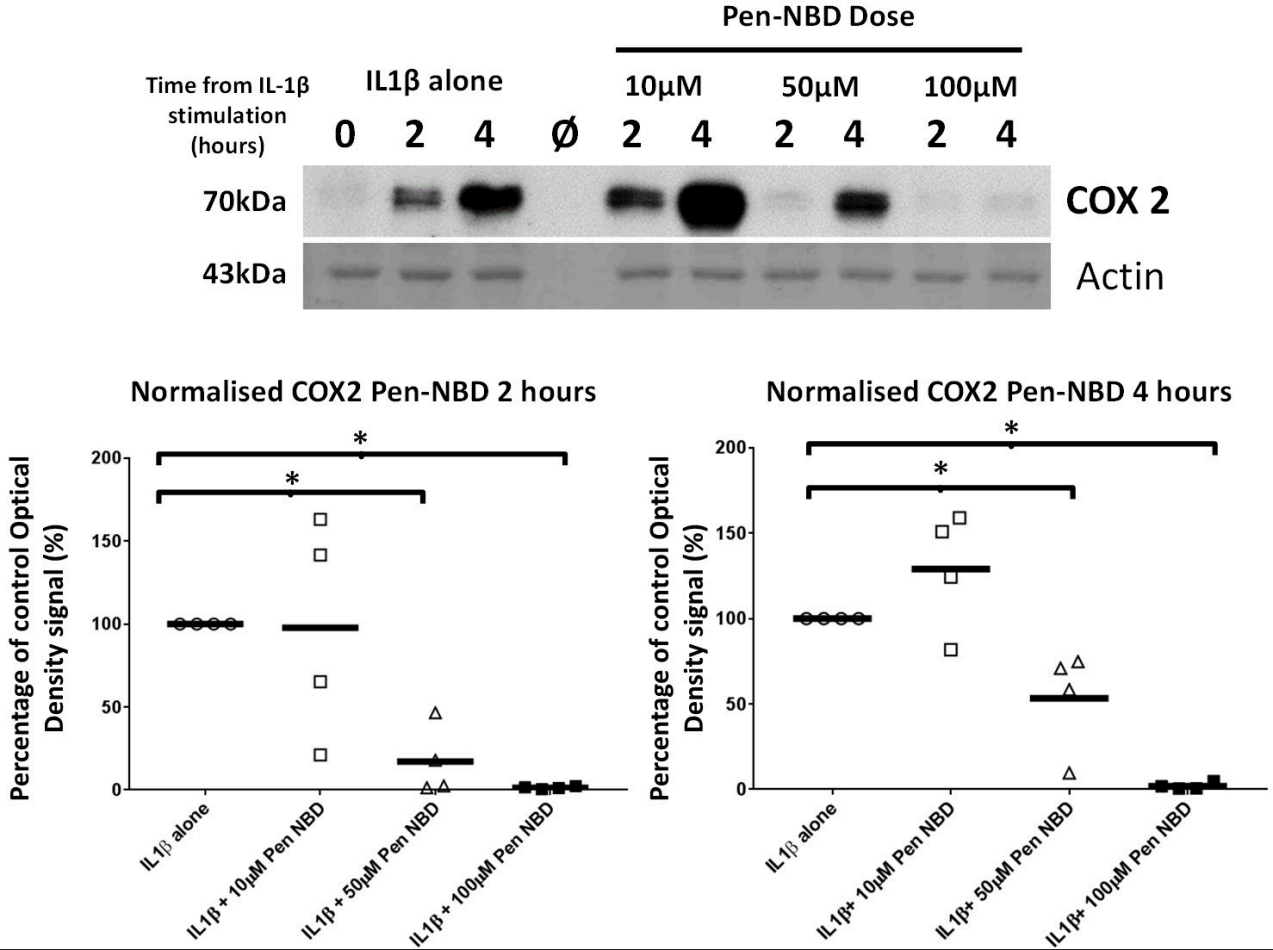
Effect of Pen-NBD peptide on IL1β-stimulated COX2 protein increases. **(Upper)** Representative Western blot demonstrating COX2 protein increase at 2 and 4 h following application of 10 ng/ml IL1β alone or with pre-incubation for 1 h with indicated concentrations of Pen-NBD peptide prior to IL1β addition. Actin expression displayed as loading control. Ø = no protein loaded. **(Lower)** Scatter plot demonstrating optical density values of COX2 protein signal at 2 and 4 h time points comparing IL1β alone or Pen-NBD plus IL1β experiments. Data presented has been normalized to control signal to demonstrate inhibitory effect. *Significant difference from raw optical density values between IL1β alone and IL1β plus Pen-NBD groups (*n* = 4, one-way ANOVA with Bonferroni *post-hoc* correction).

Initial experiments determined that CPP use was not toxic to myometrial cells (Supplementary Figure 4). Penetratin conjugated to the NBD peptide (Pen-NBD) was applied to myometrial cell cultures in increasing concentrations 1 h prior to addition of 10 ng/ml of IL1β. As demonstrated in the Western blot portrayed in Figure 2: in the absence of inhibitor (IL1β alone) induction of the protein enzyme COX2 is evident at 2 h and continues to increase at 4 h following cytokine application. Pre-incubation of 50 and 100 μM Pen-NBD led to significant inhibition of these cytokine-stimulated increases in COX2 protein signal at both 2 h (50 μM *p* = 0.01; 100 μM *p* = 0.003) and 4 h (50 μM *p* = 0.049; 100 μM *p* = 0.0003).

**Figure 3:**
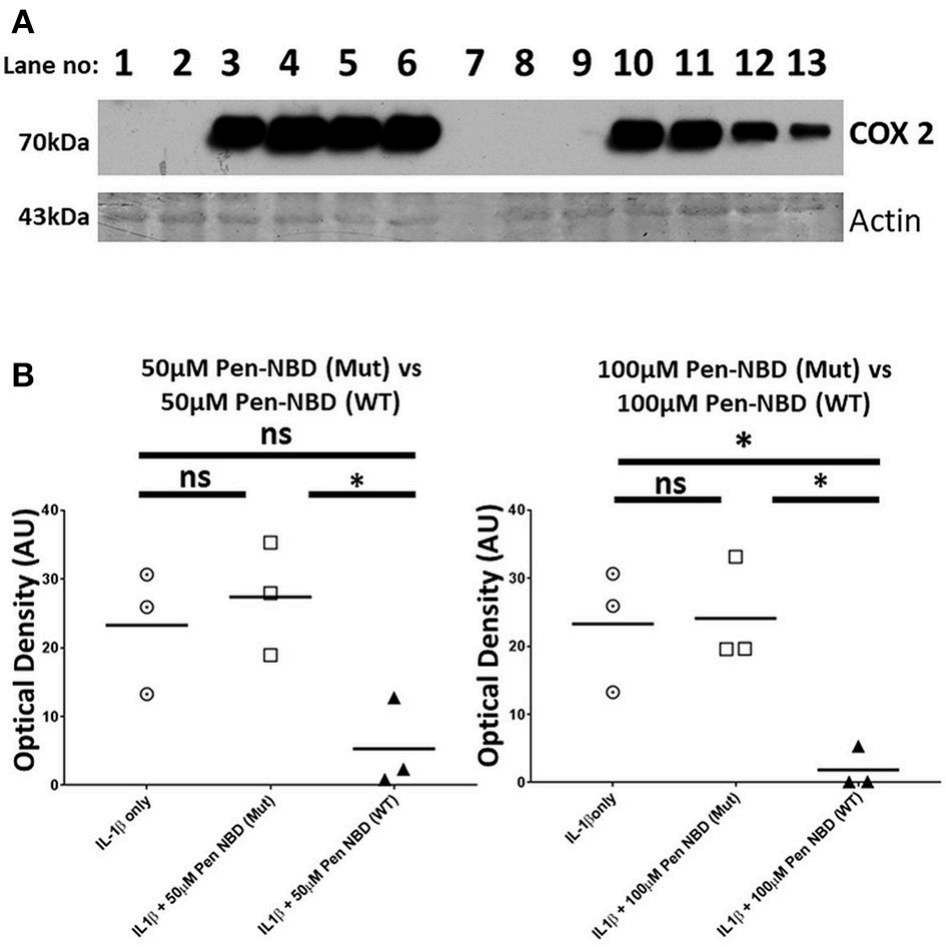
Comparison of effect of Pen-NBD mutant (Mut) peptide with Pen-NBD wild type (WT) on IL1β-stimulated COX2 protein responses. **(A)** Representative Western blot demonstrating COX2 protein changes at indicated time points. Lanes 1,2,8, and 9 display protein expression with no agonist addition. Lanes 3 and 10 display protein expression with addition of 10 ng/ml IL1β alone. Lanes 4 and 11 display protein expression with DMSO vehicle plus IL1β. Lanes 5 and 6 display protein expression with Pen-NBD (Mut) at indicated concentrations plus IL1β and lanes 12 and 13 display Pen-NBD (WT) at indicated concentrations plus IL1β. Actin expression displayed as loading control. Lane 7—no protein addition. **(B)** Scatter plots demonstrating comparison of optical density values of COX2 protein signal between IL1β only, IL1β plus Pen-NBD (Mut), or IL1β plus Pen-NBD (WT) at indicated concentrations. *Significant difference from raw optical density values between IL1β only and IL1β plus Pen-NBD groups (*n* = 3, one-way ANOVA with Bonferroni's *post-hoc* correction).

Control experiments were performed to test the specificity of COX2 responses to the NBD peptide. The inhibitory effect of Pen-NBD on IL1β-induced COX2 protein expression was compared with a Penetratin-conjugated mutant version of the NBD peptide: Pen-NBD (Mut), and NBD peptide alone (without conjugation to CPP). Figure 3 displays a representative Western blot with scatter graphs demonstrating three independent experiments to compare COX2 protein responses to IL1β following the pre-incubation with either Pen-NBD mutant or wild type peptide. It demonstrates that the mutant peptide does not diminish IL1β-stimulated COX2 induction when used at identical concentrations to the wild type peptide.

Application of non-conjugated NBD peptide had no inhibitory effect on cytokine-stimulated COX2 protein increases, nor did application of CPP alone (Supplementary Figure 1), thus indicating the requirement of CPP-conjugation to NBD for the inhibitory effect to be evident.

### In Human Myometrial Cells Pen-NBD Does Not Prevent the Cytokine-Stimulated Degradation of IκBα but Does Diminish Phosphorylation of P65 Following IL1β Exposure

It was important to assess the effect of CPP-based inhibition on protein expression changes within the canonical NFκB pathway subsequent to IL1β stimulation. Within myometrial cells, increases in the phosphorylated form of the NFκB subunit p65 protein are observed at 15 min due to the action of the activated IKK complex (Supplementary Figure 2), these responses gradually diminish over the tested 4 h time frame but do not return to normal, suggestive of increased background NFκB activity following cytokine stimulation in human uterine cells over the time frame examined. Additionally, within 15 min of exposure to IL1β, there is both phosphorylation of the inhibitory IκBα protein and degradation of its native form with full return of the native form of this inhibitory protein occurring between 60 min and 2 h.

**Figure 4:**
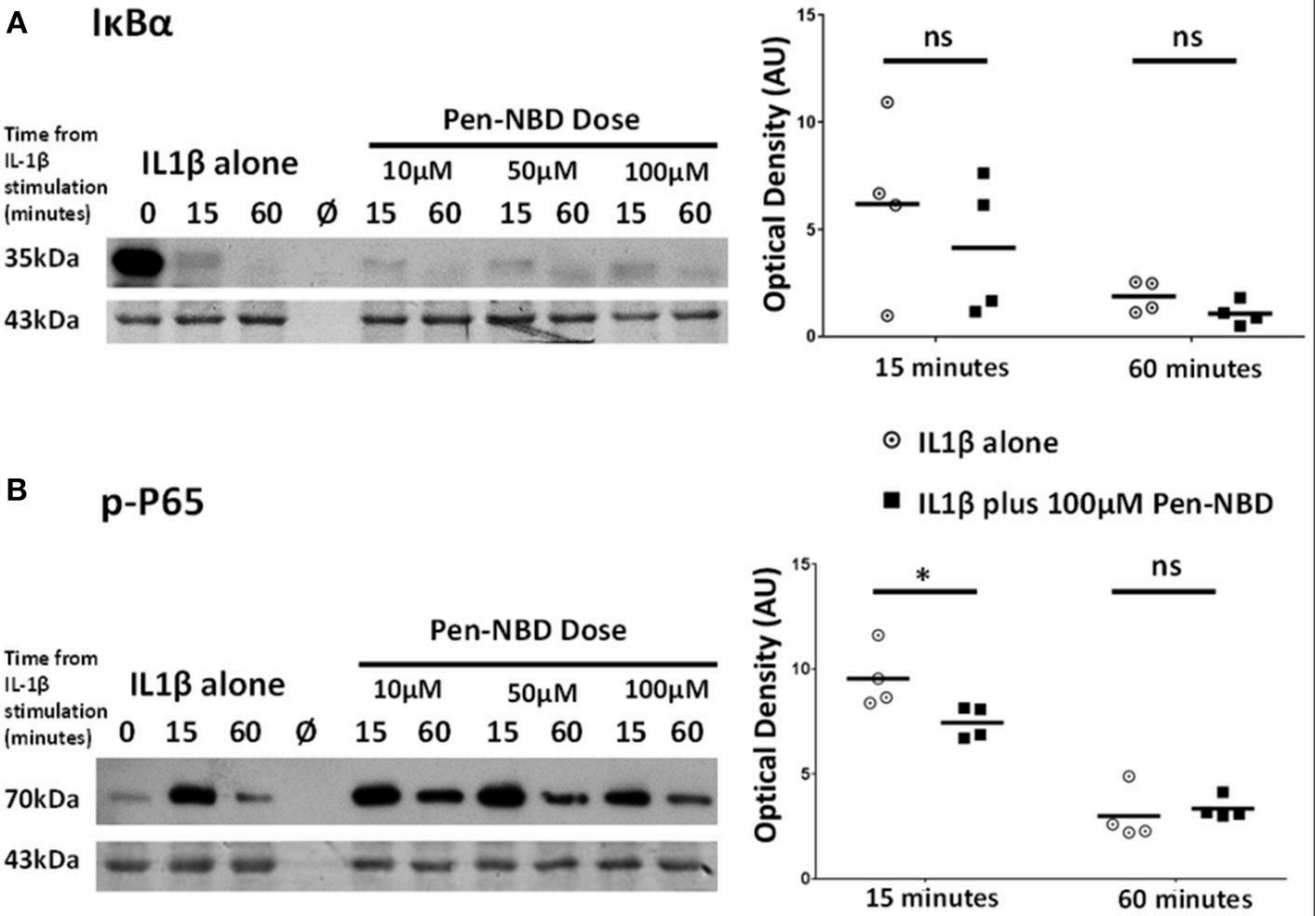
Effect of Pen-NBD on the cytokine–induced degradation of IκBα protein and phosphorylation of P65 protein. (Left) Representative Western blots demonstrating responses of **(A)** IκBα protein and **(B)** phosphorylated P65 protein at 15 and 60 min following application of 10 ng/ml IL1β either alone or following pre-incubation for 1 h with indicated concentration of Pen-NBD peptide prior to IL1β addition. Actin expression displayed as loading control. Ø = no protein loaded. (Right) Scatter plots demonstrating raw optical density values of **(A)** IκBα protein and **(B)** phosphorylated P65 protein signal at 15 and 60 min time points comparing IL1β alone or 100μM Pen-NBD plus IL1β experiments. *Significant difference from raw optical density values between IL1β alone or 100 μM Pen-NBD plus IL1β groups (*n* = 4, two-way ANOVA with Sidak's *post-hoc* correction).

At all concentrations tested, Pen-NBD did not prevent the cytokine-induced degradation of IκBα (Figure 4A). However, a significant reduction of P65 protein phosphorylation 15 min following application of IL1β was observed with the use of 100μM Pen-NBD (*p* = 0.025) (Figure 4B).

### The NBD Peptide Conjugated to the Truncated Penetratin Vector Pen(43-56) Is Capable of COX2 Inhibition but Is Less Efficacious Than Pen-NBD and Does Not Influence IKK-Dependent Protein Alterations Within the NFκB Pathway

To test the effect of alterations of the CPP vector on the efficacy of NBD cargo protein-protein interactions, experiments were performed using the NBD peptide conjugated to a truncated arrangement of the CPP Penetratin, to form Pen(43-56)-NBD. This CPP-peptide fusion has previously demonstrated ability to block IL1β-induced NFκB activity in HeLa cell lines (38), but has not been examined previously in a gestational cell setting.

**Figure 5:**
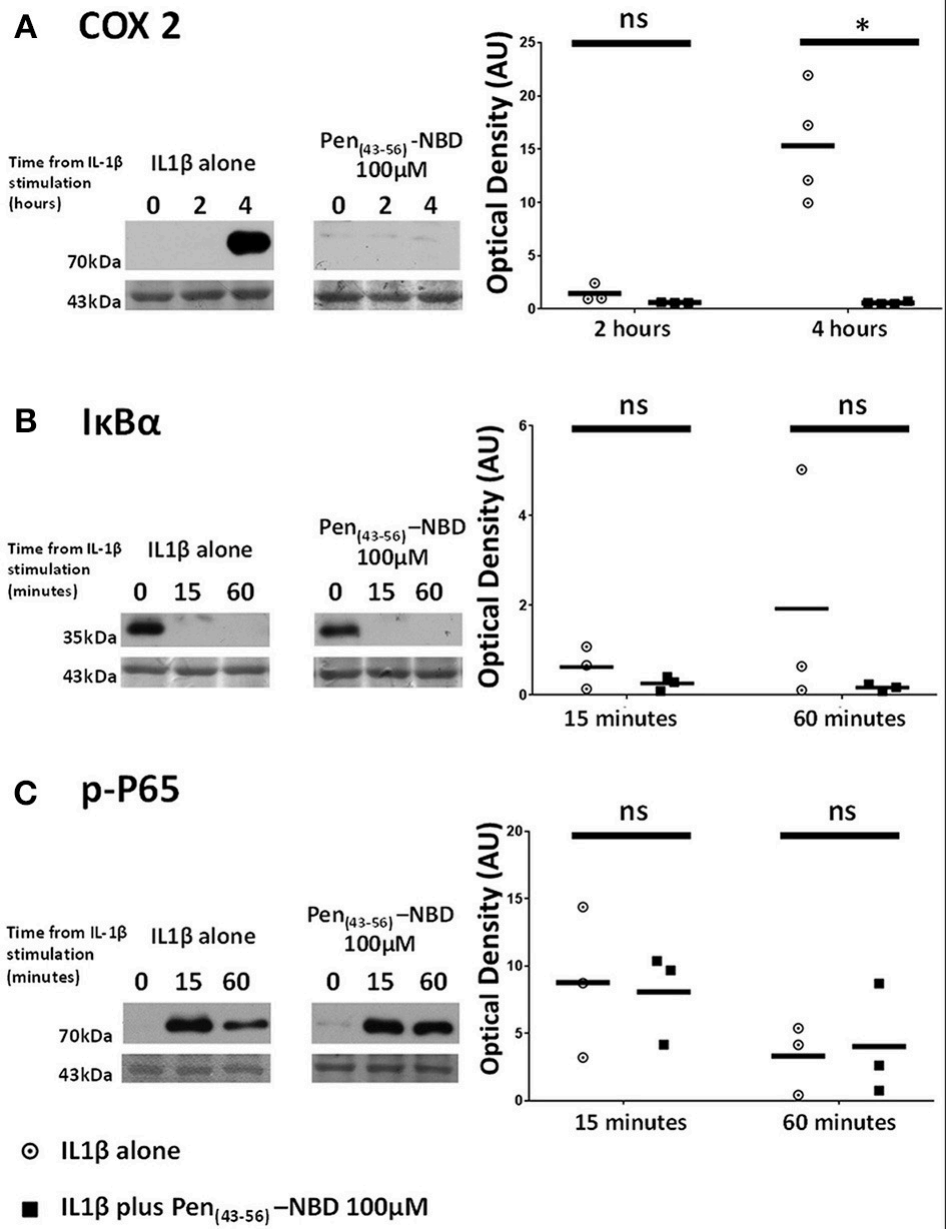
Effect of Pen (43-56)-NBD on cytokine-induced alterations of **(A)** COX2, **(B)** IκBα, and **(C)** phosphorylated-P65 proteins. (Left) representative Western blots demonstrating protein responses at indicated time points following application of 10 ng/ml IL1β either alone or following pre-incubation for 1 h with 100 μM of Pen(43-56)-NBD peptide prior to IL1β addition. Actin expression displayed beneath as loading control. (Right) Scatter plots demonstrating protein signal raw optical density values comparing IL1β alone or 100 μM Pen-NBD plus IL1β experiments.*Significant difference from raw optical density values between IL1β alone or 100 μM Pen-NBD plus IL1β groups (*n* = 3–4, one-way ANOVA with Bonferroni's *post-hoc* correction).

Applied to human myometrial cells 100 μM Pen (43-56)-NBD was able to inhibit IL1B-induced COX2 protein increases at 4 h (*p* = 0.0045) (Figure 5A). Such inhibition was not seen with the use of lower doses (data not shown). This CPP-cargo conjugation did not inhibit IκBα protein degradation or P65 protein phosphorylation (Figures 5B,C).

### The Small Molecule Inhibitor Sc514 Exerts Inhibitory Effect on COX2 Protein Induction Via Alteration of IKK-Dependent Events Within the NFκB Pathway

**Figure 6:**
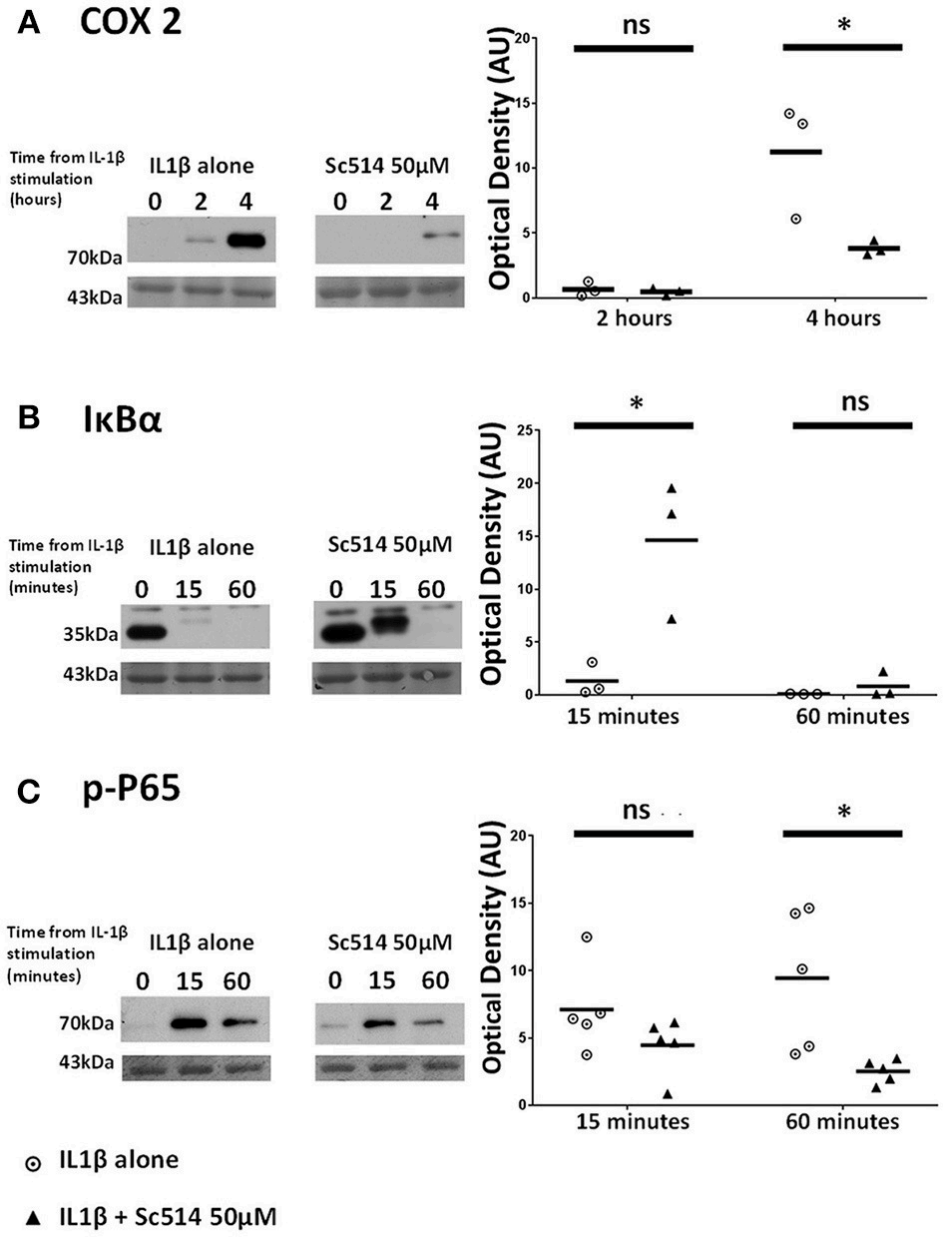
Effect of Sc514 on cytokine-induced alterations of **(A)** COX2, **(B)** IκBα, and **(C)** phosphorylated-P65 proteins. (Left) representative Western blots demonstrating protein responses at indicated time points following application of 10 ng/ml IL1β either alone or following pre-incubation for 1 h with 50 μM Sc514 prior to IL1β addition. Actin expression displayed beneath as loading control (*n* = 3 COX2/IκBα, *n* = 5 p-P65,). (Right) scatter plots demonstrating protein signal raw optical density values comparing IL1β alone or 50 μM Sc514 plus IL1β experiments. *Significant difference from raw optical density values between IL1β alone or 50 μM Sc514 plus IL1β groups (*n* = 3 COX2/IκBα, *n* = 5 p-P65 two-way ANOVA with Sidak's *post-hoc* correction).

Sc514 at 50 μM concentration was applied to myometrial cells 1 h prior to IL1β stimulation. Significant reduction in COX2 protein expression with the use of this inhibitor was observed at 4 h (*p* = 0.001). IL1β -induced degradation of IκBα was inhibited (*p* = 0.009) as was the phosphorylation of P65 protein (*p* = 0.001) (Figures 6A–C).

### IL1β Promotes Inflammation- and Labor-Related Gene Expression Changes in Human Myometrial Cells

To further demonstrate the response of myometrial cells to inflammatory stimulation and to compare the ability of CPP based and small molecule inhibitory strategies to these responses a bespoke q-PCR array panel of 42 genes was used. The genes examined in this study are listed in Table 2. The approach to gene selection for this panel was multifactorial: a number of genes encoding for proteins associated with the physiological events of uterine contraction, cervical dilation and membrane rupture that occur during human labor, termed labor associated genes, were selected (41); also, for comparison, a collection of genes encoding for G-proteins involved in maintaining uterine quiescence (42). A number of pro inflammatory genes, including genes encoding for proteins in the NFκB pathway (16), and genes involved in the production of prostaglandins (18) were chosen to aid understanding of the potential mechanism of action of inhibitors used in this study. A selection of novel genes that demonstrate altered expression in the presence of human labor were also included in the array (43).

**Figure 7:**
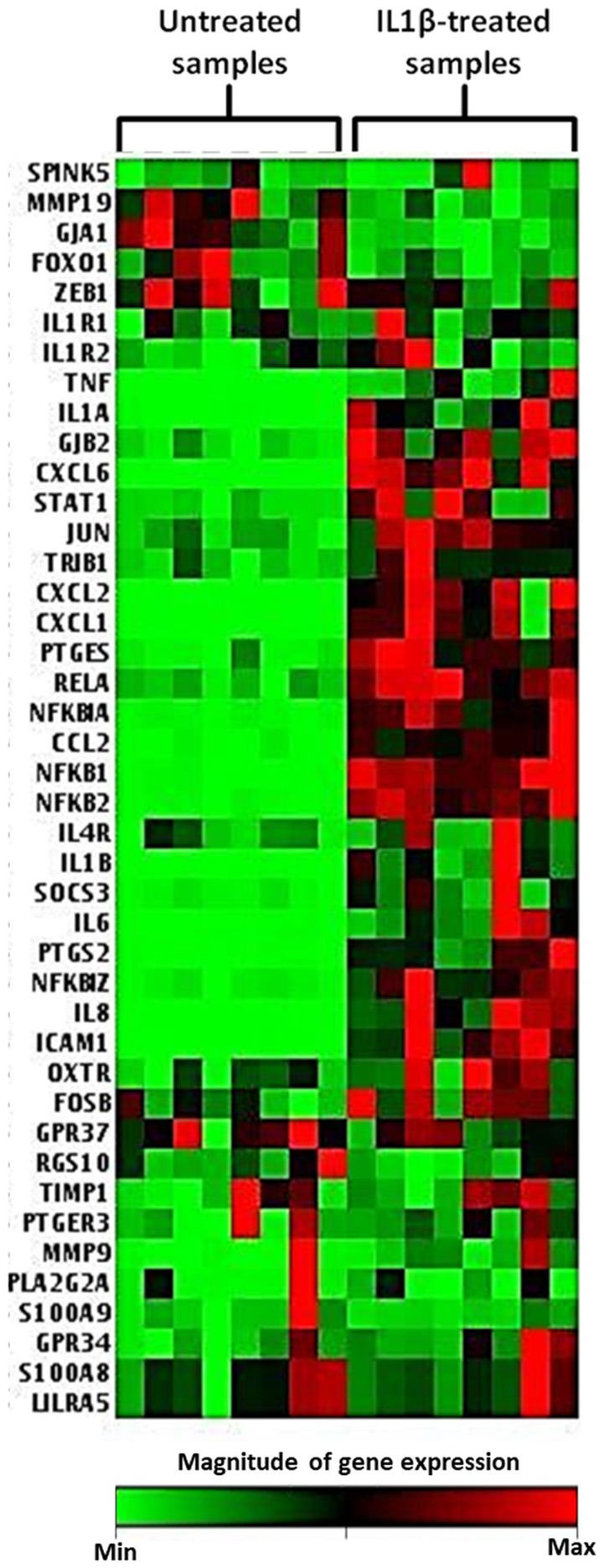
Heat map demonstrating myometrial cell gene array expression in response to IL1β cytokine stimulation. The heat map displays gene expression changes from an array of 42 GOI for untreated and IL1β treated samples from eight biological replicates. Highly expressed genes are displayed as shades of red and minimally expressed genes are shown as shades of green (*n* = 8).

Across eight independent samples a series of gene expression changes were observed in myometrial cells 4 h following exposure to 10 ng/ml IL1β. These changes included genes involved in human labor, inflammatory processes, NFκB signaling, and prostaglandin expression as demonstrated in the heat map displayed in Figure 7.

**Figure 8:**
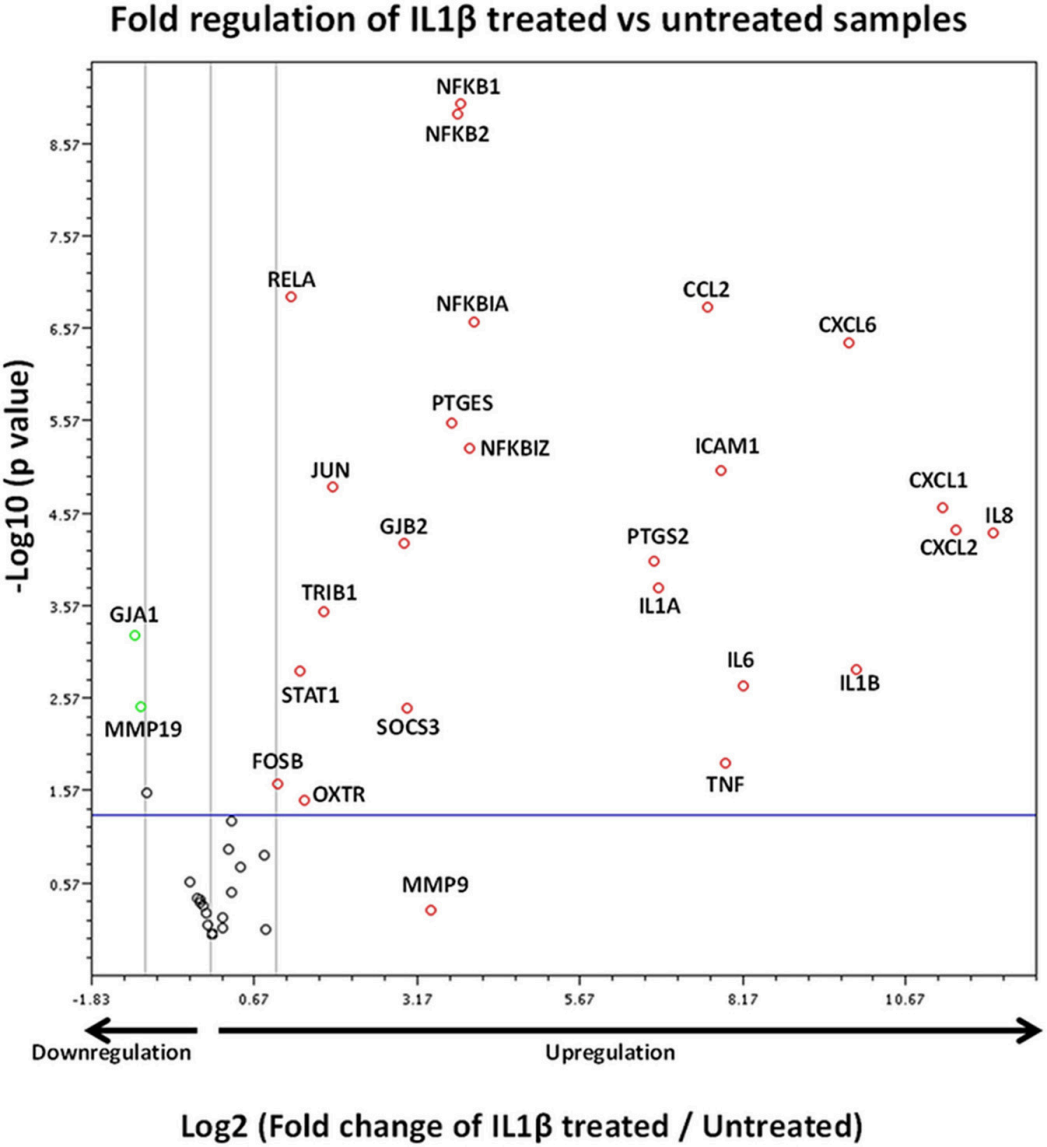
Volcano plot demonstrating gene expression changes in myometrial cells following IL1β exposure. X axis shows fold regulation changes between IL1β treated and untreated samples 4 h following cytokine addition. Y axis is the inverse of the p value generated for each gene of interest (GOI) via students *T*-test. All genes with >2 fold difference between IL1β treated and untreated samples are labeled on the figure. Upregulated genes are presented as red circles, downregulated genes are green circles and genes demonstrating no change are black circles. Genes above the blue horizontal line represent significant expression changes between IL1β and untreated samples (Student's *t*-test of the replicate 2^∧^ΔΔCt values). Data is summation of experiments from eight biological replicates (*n* = 8).

The volcano plot presented in Figure 8 demonstrates those genes exhibiting significant expression changes with greater than 2-fold difference between IL1β-treated and untreated samples. The genes displaying the largest expression changes within the panel were inflammatory cytokines/chemokines and genes associated with the NFκB family.

### CPP-Conjugated and Small Molecule Inhibitors of NFκB Dampen the IL1β-Stimulated Expression of Different Groups of Genes in Myometrial Cells

Having identified expression changes within the gene array panel following stimulation by IL1β, it was important to examine how this expression pattern would be altered using a CPP-based or small molecule inhibitory strategy. It was also of interest to observe how alterations to vector structure may affect the efficacy of CPP-cargo inhibition.

Application of either Pen-NBD, Pen(43-56)-NBD or Sc514, 1 h prior to cytokine exposure, significantly inhibited the IL1β-stimulated expression of many genes within the array panel. The pattern of inhibition can be observed in both Table 3 and the scatter graphs demonstrating fold expression changes presented within the Supplementary Data (Supplementary Figures 3A–E).

Of note, Pen-NBD and Sc514 had broad and similar anti-inflammatory effects and both approaches significantly inhibited NFκB family gene expression and prostaglandin pathway expression. For the labor associated genes: Pen-NBD, but not Sc514, inhibited the cytokine-induced expression of the oxytocin receptor gene OXTR. In contrast, Sc514, but not Pen-NBD inhibited the gene encoding for the matrix metalloproteinase MMP 9.

Use of the truncated CPP vector Pen (43-56) combined with NBD demonstrated much more limited ability to inhibit cytokine induced changes in gene expression and did not affect the expression of NFκB pathway, prostaglandin, or labor associated genes. This further demonstrates the importance of appropriate vector selection for any treatment strategy involving CPP-Cargo delivery.

## Discussion

Within the reproductive literature a substantial body of evidence places intracellular NFκB activation centrally amongst the molecular pathways associated with inflammatory preterm birth (12, 44). With this in mind, a novel approach was adopted here to investigate inhibition of inflammatory signaling in human myometrial cells. We compared two classes of agent targeting the IKK complex upstream within the NFκB pathway, aiming to diminish NFκB-dependent uterine cell inflammatory responses: CPP's conjugated to the NBD peptide and the small molecule inhibitor Sc514.

The CPP-cargo construct, Pen-NBD, was used with the aim of avoiding the need for receptor-ligand interactions on the cellular membrane and thus to precisely deliver bioactive cargo to the intended intracellular target. COX2 is an inducible rate-limiting enzyme in the production of prostaglandins in uterine cells. Prostaglandins are vital mediators in the induction of laboring processes and potent uterotonins that can initiate uterine contractions therefore the demonstration in this study that CPP mediated delivery of the NBD peptide inhibits the IL1β-induced expression of COX2 is of importance. A previous study examining the effect of the NBD peptide in a sheep model found it to be broadly ineffective at dampening inflammatory responses in sheep fetal membranes at 10 μM (45). This work supports that conclusion, but also emphasizes the importance of basic testing of novel compounds over a broad time and concentration range as inhibition of inflammatory responses was only observed at concentrations greater than or equal to 50 μM within the experiments presented here.

Hundreds of CPP sequences have now been discovered that could be linked to the NBD peptide. Even amongst well-characterized CPPs such as Penetratin or TAT differences exist within the literature as to the precise amino acid structure used (46), and differences in the position of key amino acid residues or charged groups within the CPP vector-cargo conjugate can alter the efficiency of delivery, with the possibility that this may in turn affect the biological effectiveness and targeting of the peptide (47, 48). In order to explore this effect of vector alteration on the overall efficacy of CPP-cargo combination, this study also examined NBD delivery and biological effect when combined to a truncated form of Penetratin CPP (Pen 43–56) that has two lysine amino acid residues removed from the C-terminal end of the peptide (49). Conjugated to rhodamine Pen(43-56)–NBD demonstrated ability to enter myometrial cells within a similar time-dose range to Pen-NBD, but it was notable that the intracellular appearances of fluorophore were larger, possibly due to a clumping phenomenon secondary to altered charge and pH properties of the truncated CPP-cargo construct. Pen(43-56)-NBD also required increased concentrations to inhibit cytokine-induced increases in COX2 protein when compared to the non-truncated form and did not prevent alterations of NFκB pathway proteins downstream of IKK complex activation. Additionally, pen(53-46)–NBD was less effective in attenuating IL1β-induced gene expression changes compared with Pen-NBD peptide. Thus, careful selection of the CPP vector used, consideration of vector-cargo interactions and investigation of mechanisms of action are vital considerations for work involving peptide-based therapies.

The small molecule inhibitor Sc514 was chosen to compare this CPP-based therapeutic approach as it has a putatively very similar mechanism of action focussed on interaction with IKK complex to prevent its activation within the canonical NFκB pathway. As with Pen-NBD, Sc514 demonstrated ability to inhibit cytokine-induced COX2 production and p65 phosphorylation. However, whereas Pen-NBD did not prevent the IL1β-dependent degradation of IκBα, Sc514 demonstrated ability to inhibit degradation of this protein. Therefore, the ability of Sc514 to prevent the degradation of this protein in comparison to the NBD cargo may relate to subtle differences in mechanism of action. The IKKβ subunit is primarily responsible for the phosphorylation of IκBα from which subsequent degradation occurs and Sc514 has previously demonstrated specificity toward IKKβ inhibition (35), whereas NBD peptide blocks association between NEMO and the other subunit portions of the IKK complex (29). Thus, the inability of Pen-NBD to prevent IκBα degradation in myometrial cells could either be due to an incapacity to dampen IKKβ-specific responses or to the actions of alternative IKK complexes within the cell that can lack Nemo Binding Domain (50).

To examine the wider anti-inflammatory effects of both CPP-cargo and small molecular inhibitory approaches the expression of a bespoke array of genes was used to examine myometrial gene expression changes in response to cytokine stimulation, and, subsequently, the effect of the inhibitory approaches to dampen such responses. Application of IL1β to myometrial cells produced many gene expression changes that have been previously observed in comparisons between human laboring and non-laboring samples including upregulation of NFκB pathway components, cytokines such as IL6 and IL8 and genes with specific associations with human labor such as PTGS2 and OXTR (51, 52).

Sc514 and Pen-NBD had similar anti-inflammatory effects dampening the cytokine-provoked transcription of NFκB family genes and key cytokines such as IL6 and IL8. There were interesting variations in this effect with Pen-NBD demonstrating inhibition of OXTR, the gene transcribing for oxytocin receptor protein; whereas Sc514 inhibited the cytokine-induced upregulation of the matrix metalloproteinase enzyme gene MMP 9. Such variations suggest that with future work to clarify the detailed mechanistic processes behind the varied presentations of preterm birth, different inhibitory approaches could be taken dependent on individual biological situations. It also gives rise to the possibility that in situations where a broad insult is responsible for the onset of preterm birth, more than one agent may be required to prevent this outcome.

## Limitations

A consistent limitation of fundamental biological studies investigating several outcomes of multiple interventions when using primary sourced human material is tissue availability. This can be further exacerbated when studies refrain from using cells at high passage (>4) to minimize potential deviation from original cellular phenotype. Within such practical and temporal constraints, the final numbers used in some experiments within this study are low; however, the authors feel this does not detract from the innovative nature of the study, and offers incentive for more detailed investigation of the uses of CPP based therapies and Sc514 as inflammatory and potentially contractile inhibitors for use in the field of pregnancy research.

## Conclusion

Due to the shortage of candidate agents aimed at the prevention of preterm birth available for clinical testing, a number of approaches have been investigated with the intention of dampening inflammatory-mediated signaling in uterine cells (53, 54). However, most of these, outside of anti-infection strategies, rely upon inhibiting plasmalemmal receptor engagement with cognate ligands. This study presents a novel approach to addressing this problem by establishing that a biologically active cargo, intracellularly delivered by a CPP, can inhibit inflammation-related signaling in human myometrial cells in a similar manner to that of the potent cell-permeable small molecule inhibitor Sc514.

CPPs have the potential to deliver a diverse range of cargo to cells thus the proof of concept demonstrated here that CPP-cargo constructs enter human uterine smooth muscle cells and dampen inflammatory pathways in a targeted fashion is encouraging for the further pursuit of strategies involving varied CPP-cargo constructs that could include targeting alternative inflammatory pathways associated with preterm birth such as MAP kinase and AP1 (10, 11). It is well-established that many protein-protein interactions are classed as non-druggable and cannot be inhibited with small molecule drugs or large proteins and this offers an opportunity for investigating more peptide therapeutics delivered by CPPs.

Although further extensive preclinical testing of these therapeutic strategies is required to establish safety and efficacy over a broad concentration range; the authors envisage the future usage of such agents in an acute setting for pregnant patients presenting with the clinical phenotype of regular contractions before 37 weeks of completed gestation with the intention of both delaying birth to reduce the impact of prematurity on the fetus, and ameliorating the effect of inflammatory insult to both mother and fetus. Treatment delivery could be either through local application to gestational tissues via vaginal pessary; or techniques are evolving quickly for tissue and/or cell-specific delivery of biologically active mediators, including those directed toward uteroplacental environments (55, 56), enabling the consideration of systemic usage. Thus, there is much promise for extending in the near future the use of Pen-NBD, other CPP-cargo conjugates and Sc514 to preclinical models of preterm birth.

## Author Contributions

LG produced data through experimentation and analyzed and presented data, he also wrote the manuscript and finalized the final paper for submission. JT produced data through experimentation and analyzed and presented data. W-CT provided assistance and advice regarding the use of Sc514. AJ and his group provided collaborative supervision for the use of cell penetrating peptides and live cell microscopy and edited the manuscript. SR provided clinical supervision and oversight throughout the research project, helped facilitate the collection and use of human samples and edited the manuscript. MT oversaw and provided supervision for the whole project and in depth editing and direction for the manuscript.

### Conflict of Interest Statement

The authors declare that the research was conducted in the absence of any commercial or financial relationships that could be construed as a potential conflict of interest.
