# Supplementary material for: Inhibition of Inflammatory Changes in Human Myometrial Cells by Cell Penetrating Peptide and Small Molecule Inhibitors of NFκB
Source: Front Immunol. 2018 Dec 20;9:2966. doi: 10.3389/fimmu.2018.02966 (PMC6307458; doi:10.3389/fimmu.2018.02966)
Supplement: Supplementary file 1 [file Data_Sheet_1.docx]

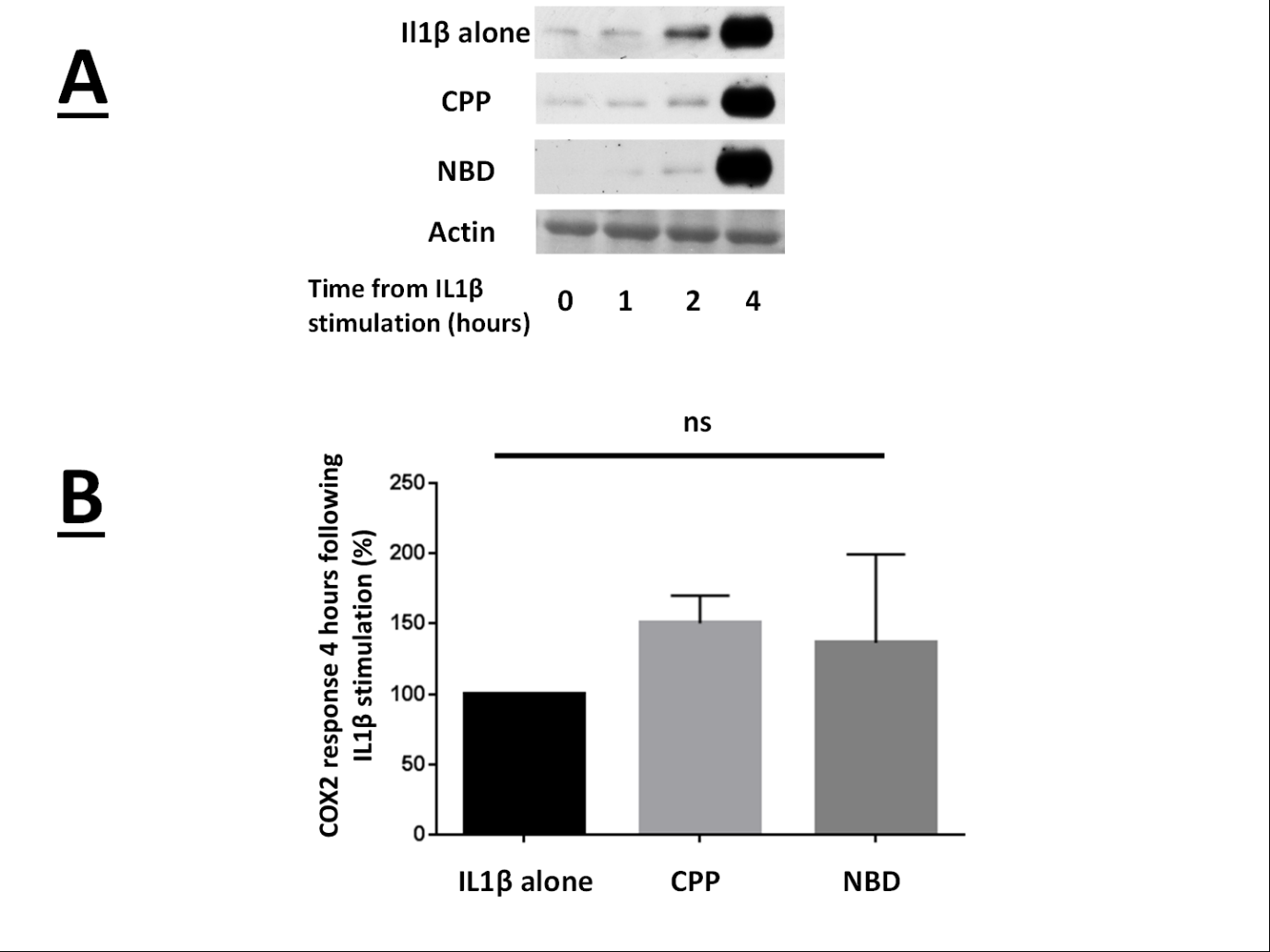


**Supplementary Figure 1** Unconjugated CPP or NBD peptide effect on IL1β-stimulated COX2 protein responses

**A** Representative Western blots of COX2 responses over a 4 hour time frame following addition of IL1β alone or IL1β plus 50μM of either unconjugated Pen(43-56) or NBD peptide. Actin expression displayed as loading control.

**B** Bar chart of mean average (SD) optical densitometry readings of 4 hour COX2 signal. Data is presented as mean (SD) average percentage values of IL1β alone (n=3).

**Supplementary Figure 2** Demonstration of effect of IL1β stimulation on phosphorylated and native forms of NFκB P65 protein in human myometrial cells over a four hour time frame.

Western blots display the protein expression of p-P65 and P65 over four hours following application of 10ng/ml IL1β. PVDF membranes stained with napthol blue-black dye demonstrate actin expression to display protein loading.

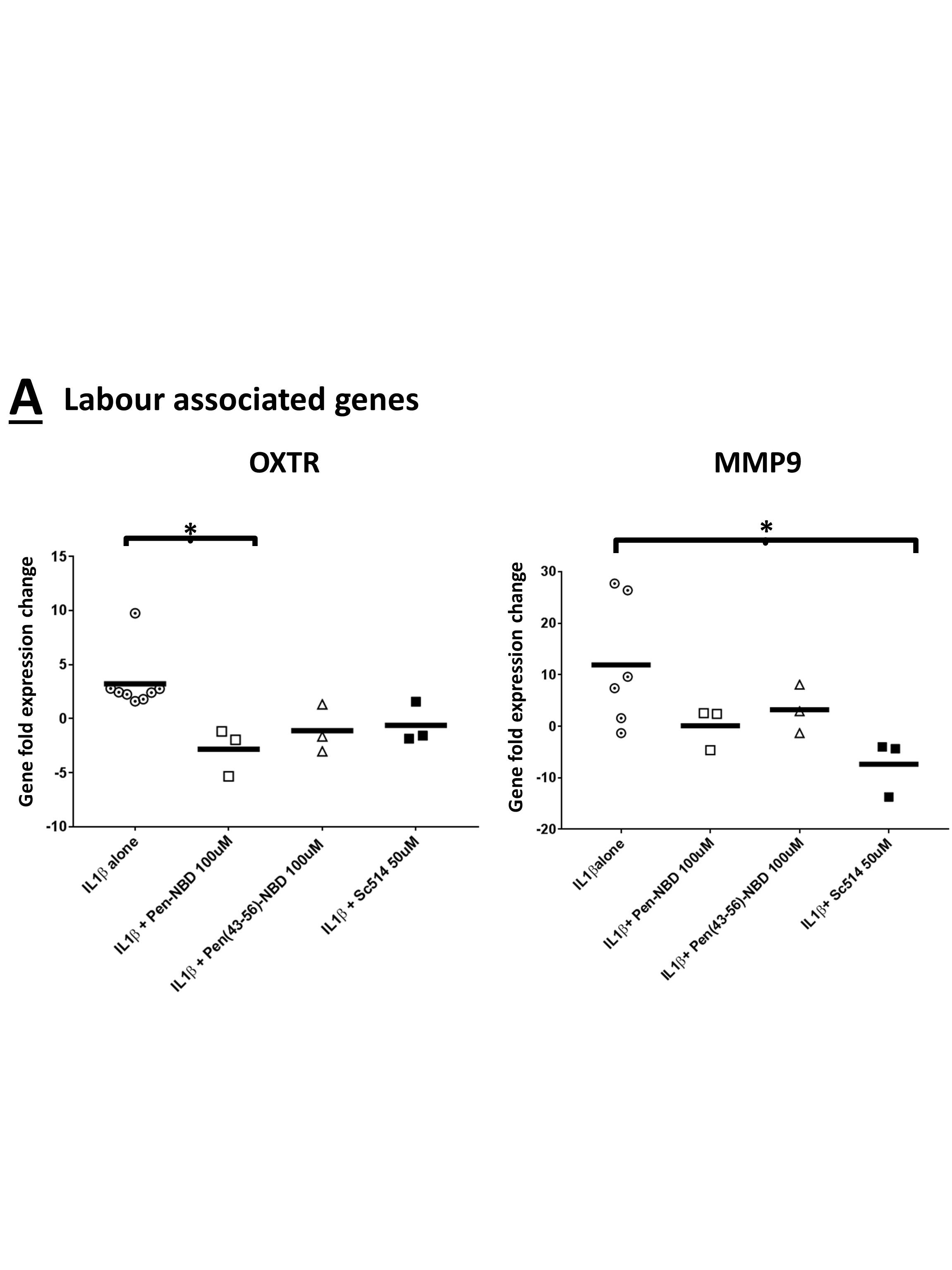


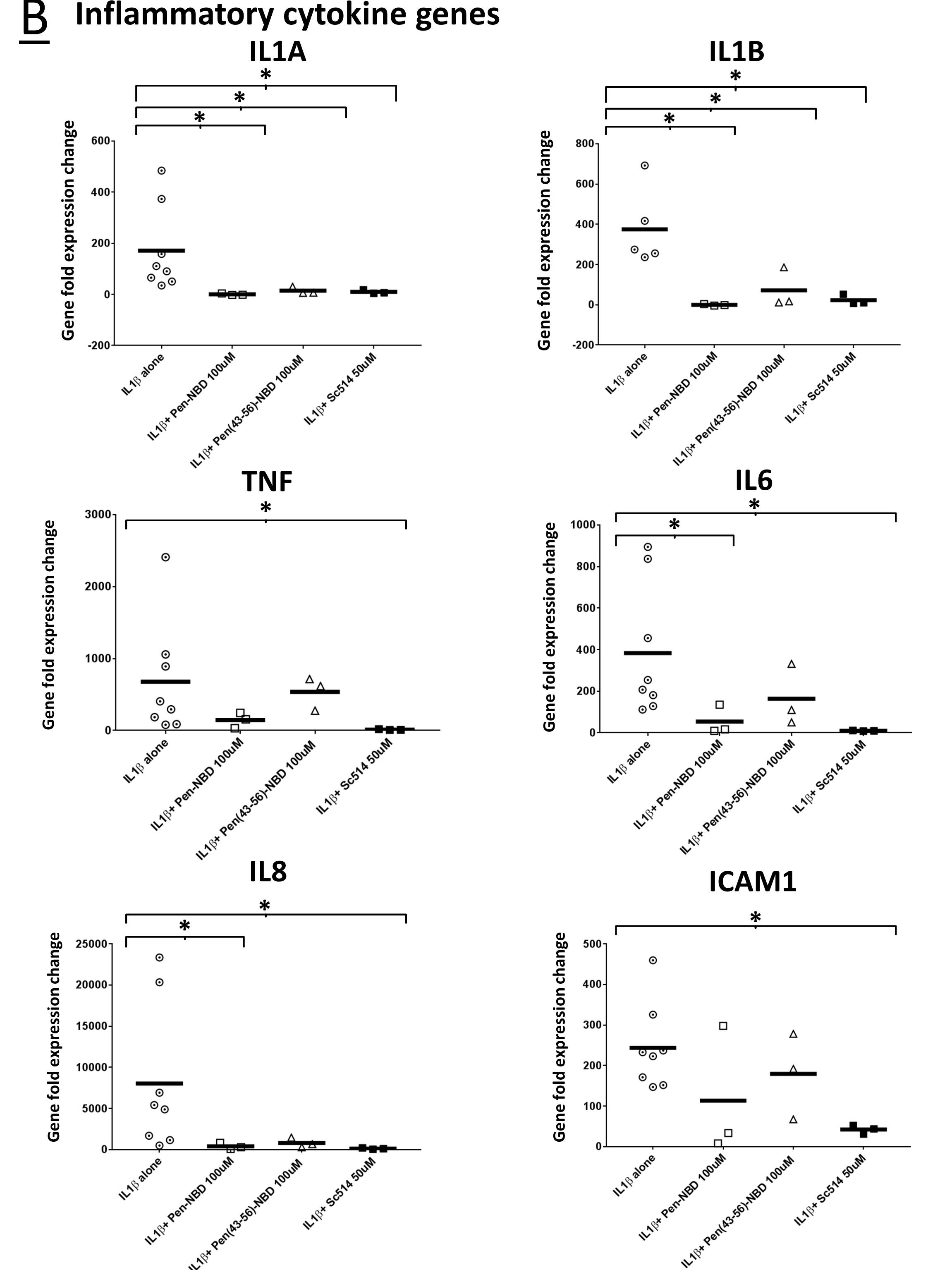


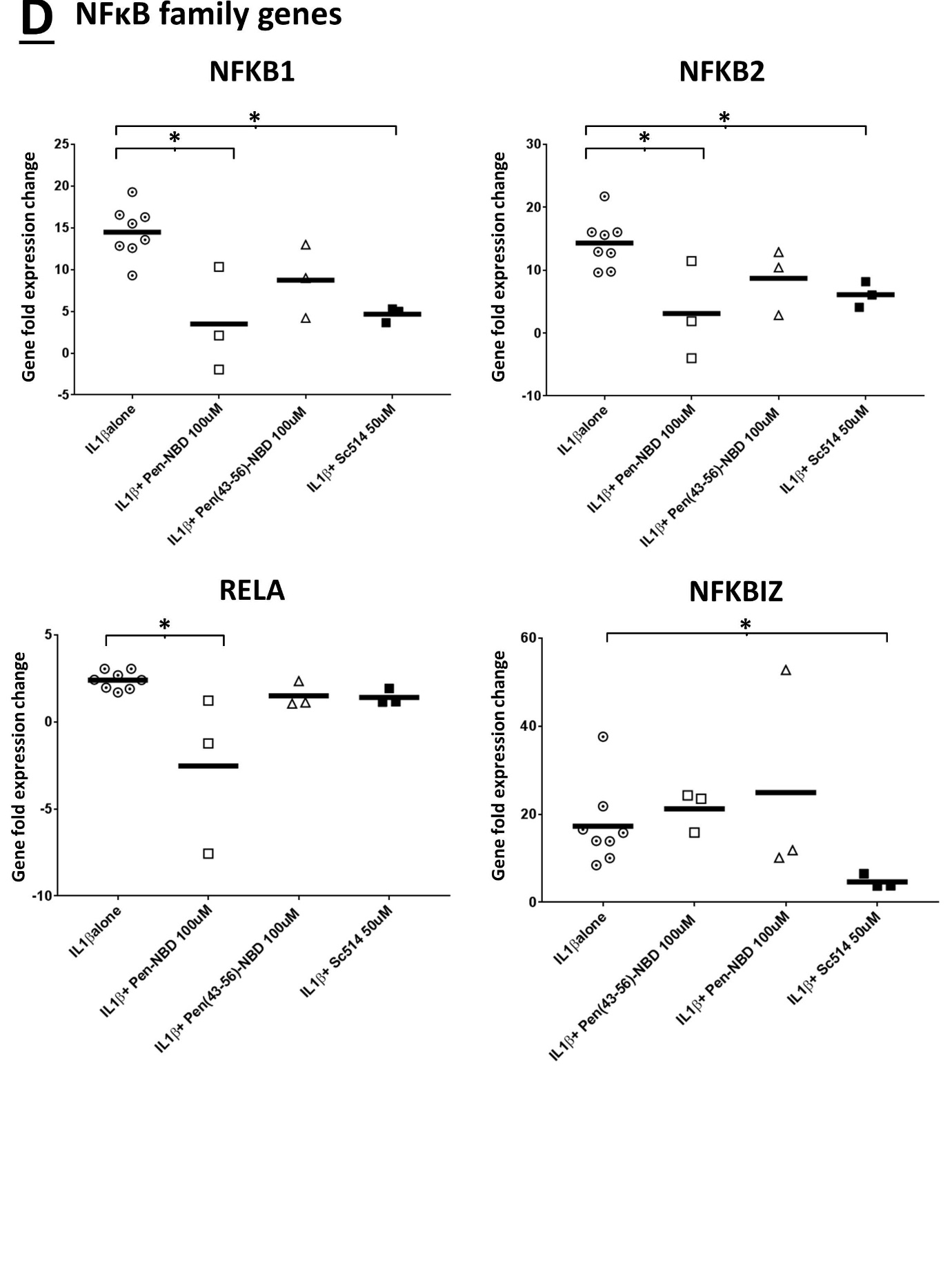


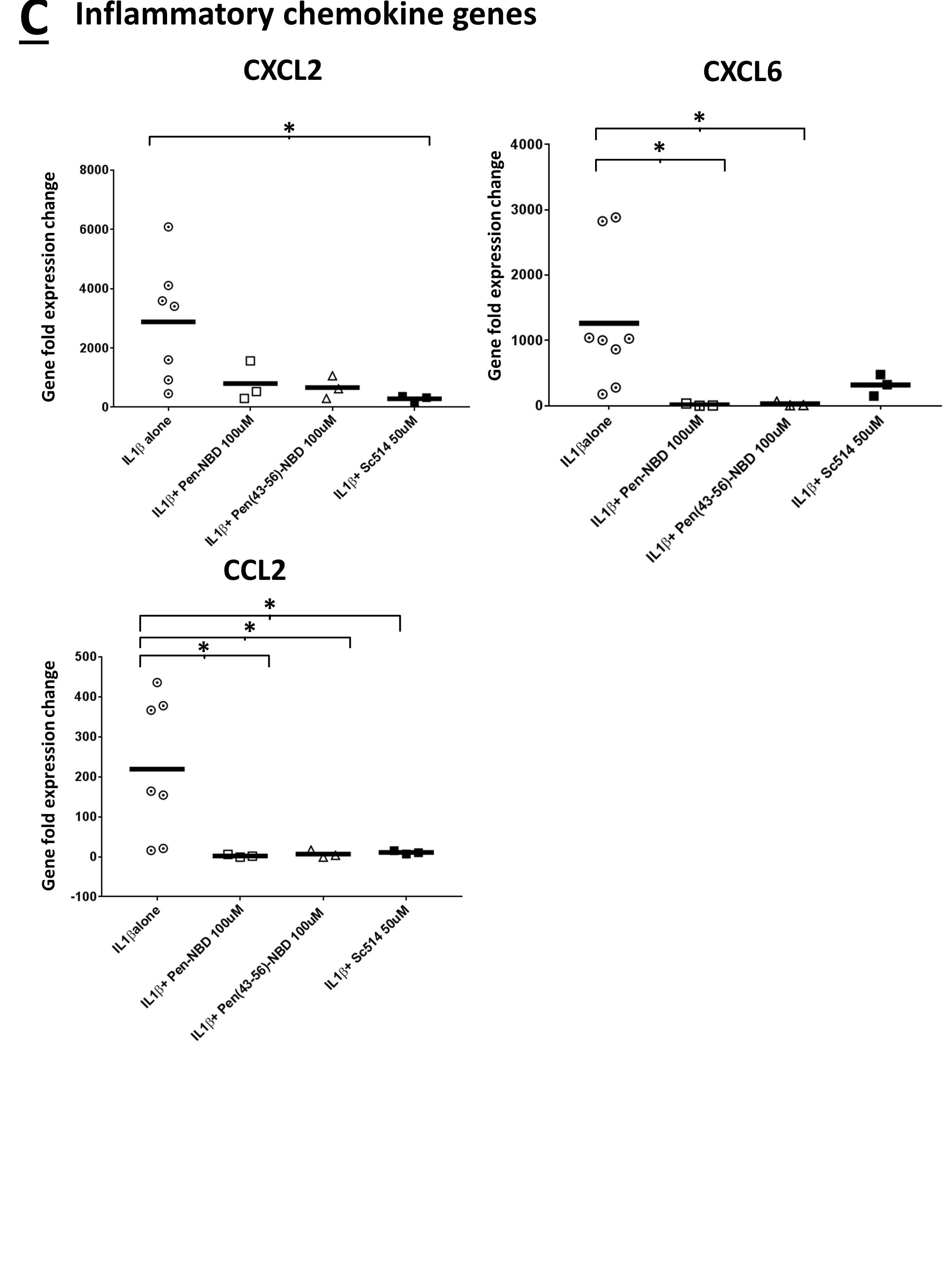


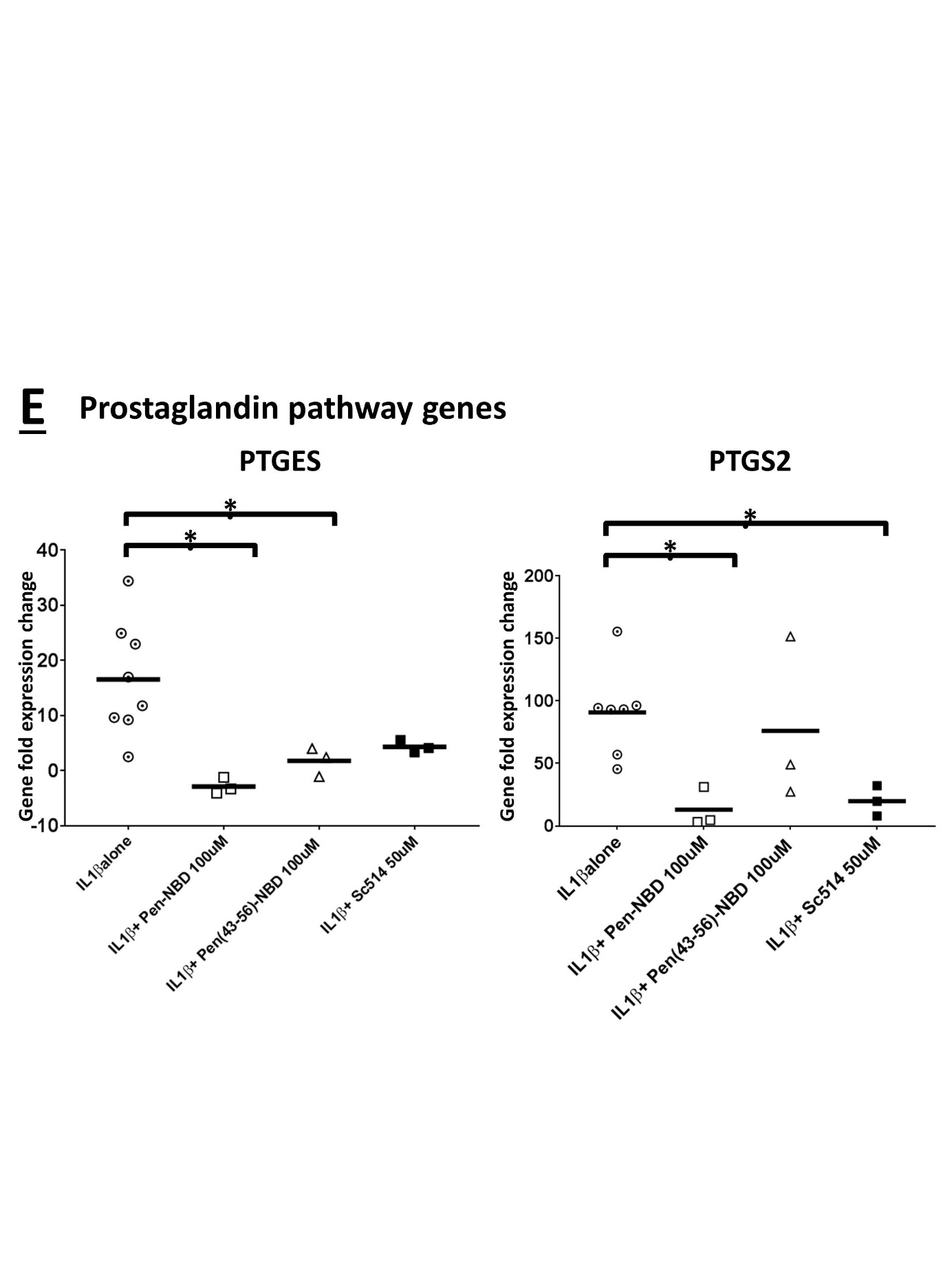


**Supplementary Figure 3** Scatter plots demonstrating the effect of CPP-based or small molecule inhibitors on IL1β- induced gene expression changes in myometrial cells on differing groups of genes from a 42 gene array. Genes displayed are those from the array that display significant expression changes in the presence of IL1β cytokine.

**A** Labour associated genes, , **B** inflammatory cytokine genes, **C** inflammatory chemokine genes, **D** NFκB pathway genes, **E** prostaglandin pathway genes.

* significant difference between IL1β alone and inhibitor groups (n=8 IL1β alone / n=3 IL1β plus inhibitor groups; one-way ANOVA with Bonferroni Post Hoc corrections)..

**Supplementary Figure 4**: Cell titre blue resazurin viability assay. Myometrial cells (<P4) were split, cells were counted and a volume equivalent to 5000 cells per well was added to each well of a 96 well plate. 24 hours later, standard media was changed to media containing unconjugated Pen, NBD peptide, or conjugated Pen-NBD. After 5 hours 20μL Cell Titre Blue was added to each well; this was left for 2 hours before reading the plate on a Tecan Fluorometer at 560nm excitation / 600nm emission. Signal was normalised to wells containing untreated cells. Bars represent mean average (SD) of raw fluorometer readings (n=3).

**A**


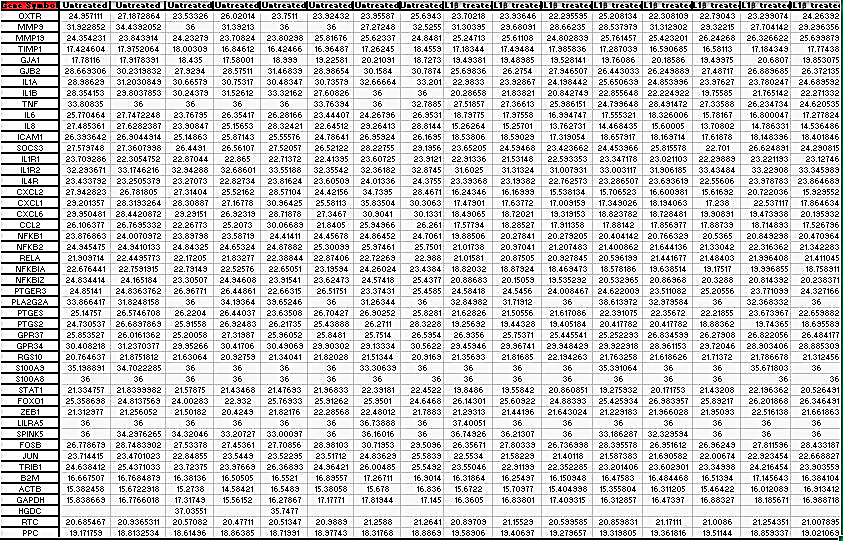


**B**


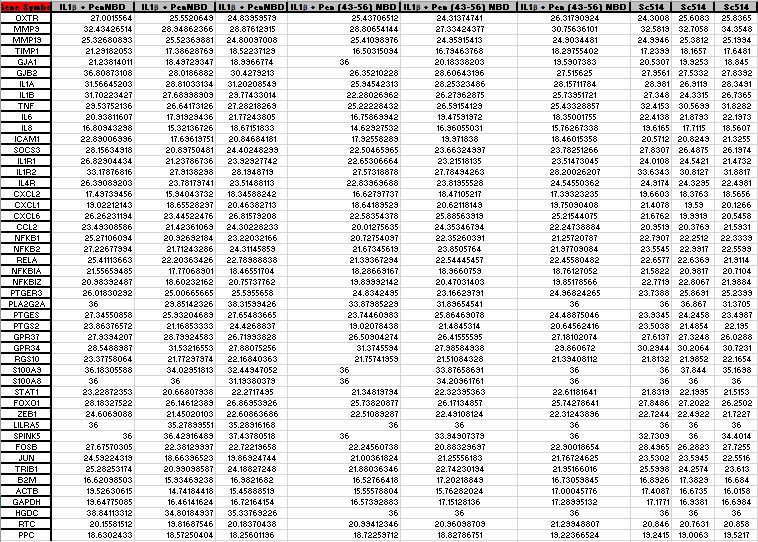


**Supplemental data** **1** Raw Ct values from qPCR array experiments

**A** Untreated samples vs IL1β treated samples

**B** IL1β plus indicated inhibitor

*NB* Values of ≥36 are indicated as 36 to allow for data comparison


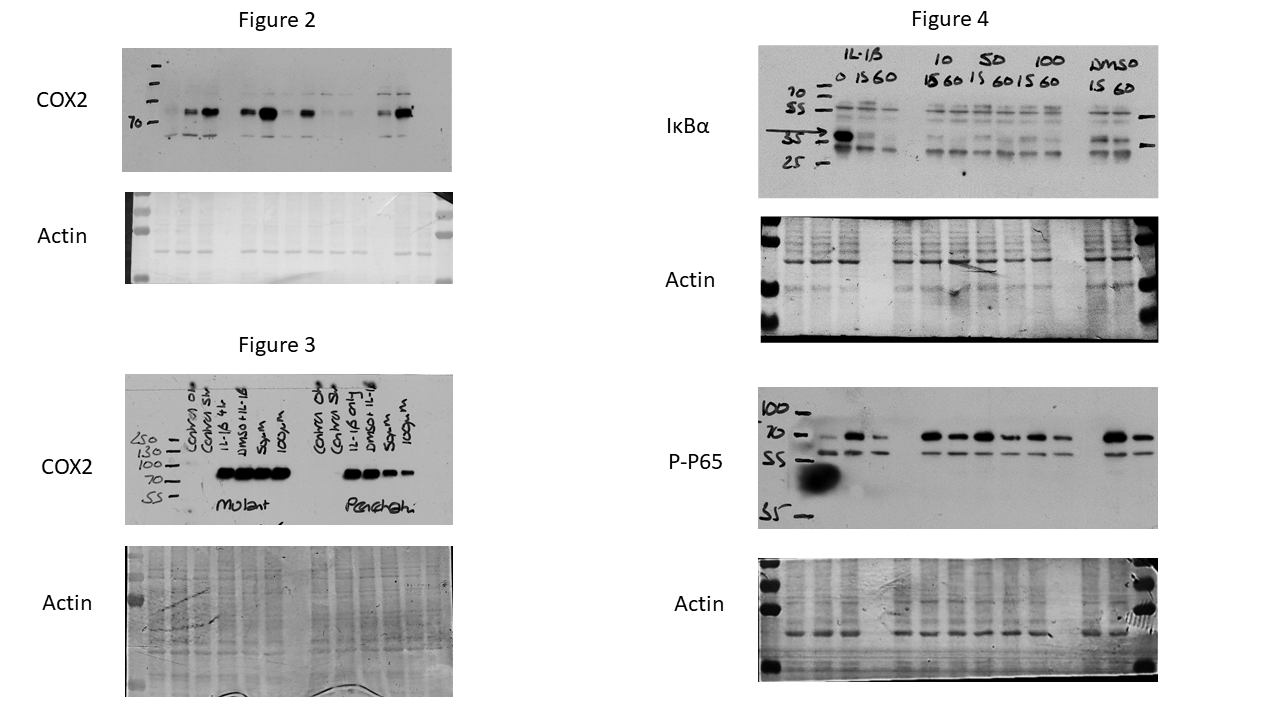


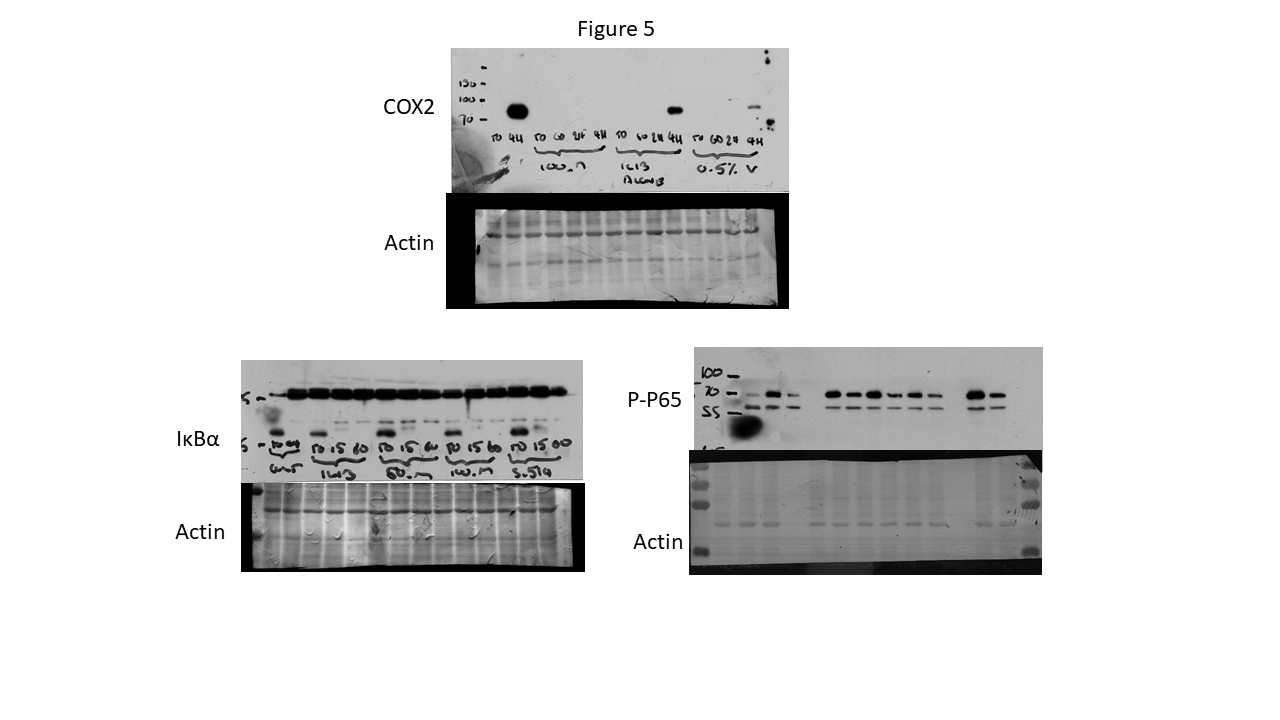


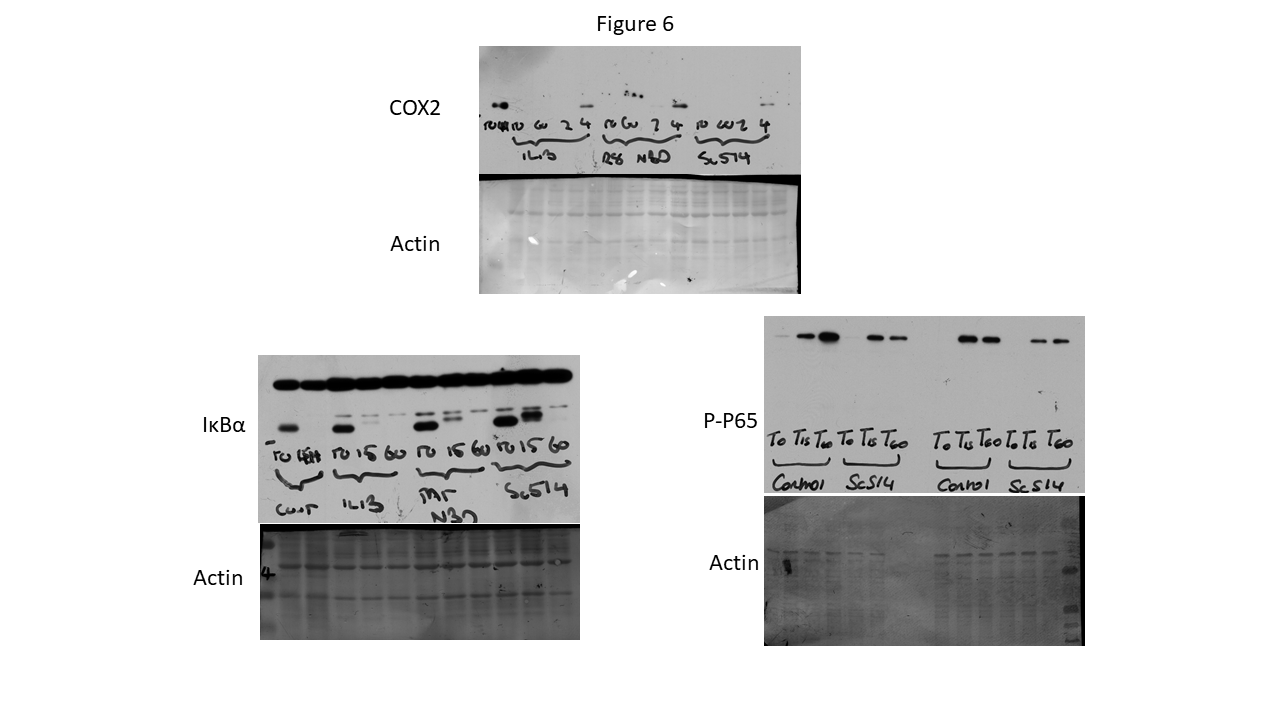


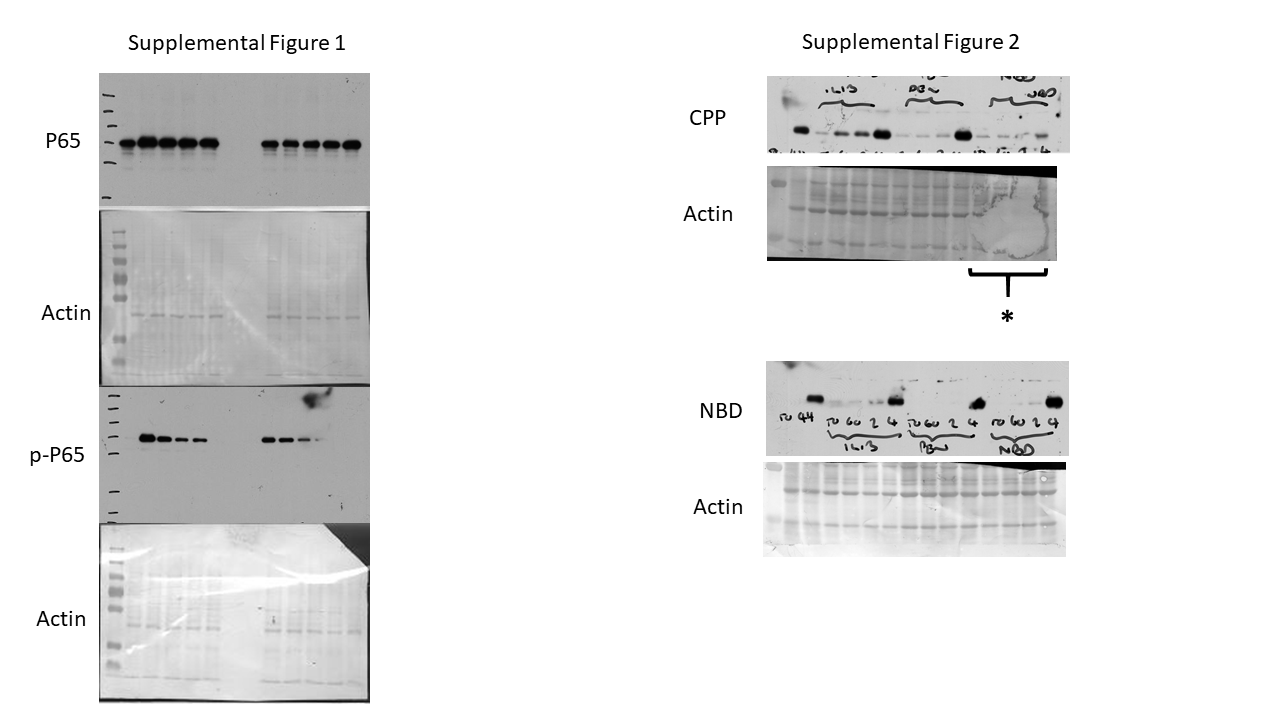


**Supplemental Data 2**: Original Western Blots and Stained PVDF Membranes

Original scanned blots from which images displayed in figures (plus supplemental figures)

* 4 lanes not used due to corrupted loading
